# Role of Helical Structure in MBP Immunodominant Peptides for Efficient IgM Antibody Recognition in Multiple Sclerosis

**DOI:** 10.3389/fchem.2022.885180

**Published:** 2022-06-20

**Authors:** Agnieszka Staśkiewicz, Michael Quagliata, Feliciana Real-Fernandez, Francesca Nuti, Roberta Lanzillo, Vincenzo Brescia-Morra, Hendrik Rusche, Michal Jewginski, Alfonso Carotenuto, Diego Brancaccio, Rina Aharoni, Ruth Arnon, Paolo Rovero, Rafal Latajka, Anna Maria Papini

**Affiliations:** ^1^ Interdepartmental Research Unit of Peptide and Protein Chemistry and Biology, Department of Chemistry “Ugo Schiff”, University of Florence, Sesto Fiorentino, Italy; ^2^ Department of Bioorganic Chemistry, Faculty of Chemistry, Wroclaw University of Science and Technology, Wroclaw, Poland; ^3^ Multiple Sclerosis Clinical Care and Research Centre, Department of Neurosciences, Reproductive Sciences and Odontostomatology, University of Naples “Federico II”, Naples, Italy; ^4^ Fischer Analytics GmbH, Weiler, Germany; ^5^ CY PeptLab Platform of Peptide and Protein Chemistry and Biology and UMR 8076 CNRS-BioCIS, CNRS, CY Cergy Paris Université, Neuville sur Oise, France; ^6^ Department of Pharmacy, University of Naples “Federico II”, Naples, Italy; ^7^ Department of Immunology, The Weizmann Institute of Science, Rehovot, Israel; ^8^ Interdepartmental Research Unit of Peptide and Protein Chemistry and Biology, Department of NeuroFarBa, University of Florence, Sesto Fiorentino, Italy

**Keywords:** multiple sclerosis, circular dichroism, immune response, synthetic helical peptides, myelin basic protein antigen, peptide-antigen based ELISA, NMR

## Abstract

The involvement of Myelin Basic Protein (MBP) in Multiple Sclerosis (MS) has been widely discussed in the literature. This intrinsically disordered protein has an interesting α-helix motif, which can be considered as a conformational epitope. In this work we investigate the importance of the helical structure in antibody recognition by MBP peptides of different lengths. Firstly, we synthesized the peptide MBP (81–106) (1) and observed that its elongation at both N- and C-termini, to obtain the peptide MBP (76–116) (2) improves IgM antibody recognition in SP-ELISA, but destabilizes the helical structure. Conversely, in competitive ELISA, MBP (81–106) (1) is recognized more efficiently by IgM antibodies than MBP (76–116) (2), possibly thanks to its more stable helical structure observed in CD and NMR conformational experiments. These results are discussed in terms of different performances of peptide antigens in the two ELISA formats tested.

## 1 Introduction

Multiple Sclerosis (MS) is a demyelinating disease of the central nervous system (CNS). Genetic and environmental factors, such as bacterial or viral infections, are involved in its multifactorial etiology. Among the viral agent putatively associated with the disease, Epstein-Barr virus (EBV) has been repeatedly reported (Meier et al., 2021; Robinson and Steinman, 2022), and the *Haemophilus influenzae* has been described as possible bacterial triggering agent in MS (Walvoort et al., 2016). Although MS etiology and pathogenesis are not fully clarified, it is widely accepted that a T-cell-mediated inflammatory process directed against myelin and other related proteins plays a crucial role, jointly with a possible role of B cells (Steinman, 1996; Rahmanzadeh et al., 2018; Díaz et al., 2019). Recent discoveries suggest that B lymphocytes substantially contribute to its initiation and chronic propagation. The fulminant clinical success of anti-CD20 antibodies in the treatment of MS, raised awareness that besides T-cells, B cells play a decisive role in MS. Synergic interaction of B and T-cell mechanisms were recently implicated as possible causes of MS (van Langelaar et al., 2020).

Due to its heterogeneity, no fully specific MS biomarkers are available, thus hampering both diagnosis and patient stratification. Considering that recent studies on B cells demonstrated the critical role of antibodies in MS pathology, identification of the antigenic targets recognized by specific antibodies in MS patient sera may help in the patient stratification process.

One of the most deeply studied myelin antigens related to MS is the myelin basic protein (MBP), a 170 amino-acid protein, which is the second most abundant component of the myelin sheath and plays an essential role in the myelination process (Moscarello, 1997; Lolli et al., 2006). Moreover, MBP is involved in the adhesion of the cytosolic surfaces of multilayered compact myelin, interacting with several polyanionic proteins, including actin, tubulin, Ca^2+^-calmodulin, and clathrin, and with negatively charged lipids, thus changing its structure upon binding to them (Boggs, 2006).

Although MBP has not been demonstrated to be the main autoantigen in MS, MBP-specific autoreactive T-cells have been found in blood of MS patients at a higher rate than in healthy individuals (Tejada-Simon et al., 2003). Moreover, immunization of susceptible mouse strains with MBP induces a T-cell response that causes experimental autoimmune encephalomyelitis (EAE), considered a valuable mouse model of human MS (Zamvil and Steinman, 1990). These evidences suggest that MBP may be a candidate autoantigen in MS. In fact, it has been reported that autoantibodies from MS patient sera recognize MBP and recruit inflammatory cells to focal areas, thereby targeting CNS myelin components and affecting their stability (Rozenblum et al., 2014). In this context, investigations of anti-MBP antibodies in MS have been largely described, reporting controversial results ranging from 0 to 100% of seropositivity (Cruz et al., 1987; Link, 1997). In particular, relapsing-remitting MS patients have been described to be seropositive to anti-MBP (84–100) antibodies (Lolli et al., 2005). Anti-MBP antibodies have been detected not only in sera, but also in the cerebrospinal fluid (CSF) (Warren et al., 1995; Sellebjerg et al., 2000) in 85% relapsing-remitting MS patients and 45% MS patients, respectively, compared to 2% in non-MS controls. In any case, anti-MBP antibodies and their pathogenic role have been a matter of debate for many years, while the role of myelin-reactive autoantibodies appears more defined (Martinsen and Kursula, 2021).

This discussion may be triggered by several factors, sometimes involving unclear changes at the molecular level in the composition and structure of MBP isoforms, as well as in compact myelin, during the pathogenesis of MS (Beniac et al., 1999; Boggs et al., 1999). The hypothesis of an abnormal isoform composition of MBP, leading to weakened membrane interactions and loosening the rigid myelin structure, could lead to the observed anti-MBP immune-reactivity, as well as to the presence of MBP in the CSF. Then, structural changes in the MBP sequences used in immunosorbent assays could explain, at least in part, not only the controversial results reported about anti-MBP antibody recognition in sera samples, but also their described low affinity. In fact, cells secreting high-affinity anti-myelin antibodies have been described in CSF from MS patients, particularly when compared to circulating anti-MBP antibodies, which showed low affinity (Sellebjerg et al., 1995; O’Connor et al., 2003). In any case, the association of anti-MBP antibodies with earlier and more frequent disease relapses (Berger et al., 2003) highlights their interesting role in MS.

With all these considerations in mind, we focused our research on a further investigation of the factors involved in antibody recognition of MBP peptide antigens by MS patients. Herein, we present a study on the role of the sequence and structure of synthetic MBP peptides that have been used to identify specific antibodies in Multiple Sclerosis patient sera.

## 2 Materials and Methods

### 2.1 Reagents

All Fmoc-protected amino acids, *N*,*N*′-diisopropylcarbodiimide (DIC), OxymaPure® (ethyl-2-cyano-2-(hydroxyimino)acetate), and Fmoc-Lys(Boc)-Wang Tentagel^®^ resin were purchased from Iris Biotech GmbH (Marktredwitz, Germany). Tentagel^®^ S RAM was purchased from Rapp Polymere (Tuebingen, Germany). Peptide-synthesis grade *N*,*N′*-dimethylformamide (DMF) and acetonitrile (ACN) were purchased from Carlo Erba (Milan, Italy). Dichloromethane (DCM), trifluoroacetic acid (TFA), triisopropylsilane (TIS), and piperidine were purchased from Sigma-Aldrich (Milan, Italy). 2,2,2-trifluoroethanol (TFE) was purchased from Alfa Aesar (Kandel, Germany). Myelin Basic Protein (MBP) was purchased from Merck (Milan, Italy).

### 2.2 Microwave-Assisted Solid Phase Peptide Synthesis

Peptides 1-6 were synthesized in solid-phase using a microwave-assisted protocol (MW-SPPS) on a Liberty Blue™ automated peptide synthesizer (CEM Corporation, Matthews, NC, United States), following the Fmoc/tBu strategy as previously described (Rizzolo et al., 2011; Pandey et al., 2013; Nuti et al., 2020). Tentagel® S RAM resin (loading 0.23 mmol/g) was used for the synthesis of peptides 1, 5, and 6. Fmoc-Lys(Boc)-Wang Tentagel® resin (loading 0.23 mmol/g) was used for the synthesis of peptides 2, 3, and 4. Fmoc-deprotections were performed with a solution of 20% (v/v) piperidine in DMF (2 M). Peptide assembly was performed by repeating the standard MW-SPPS coupling cycle for each amino acid, using Fmoc-protected amino acids (2.5 equiv, 0.4 M in DMF), OxymaPure® (2.5 equiv, 1 M in DMF), and DIC (2.5 equiv, 3 M in DMF). Uncertain peptide coupling steps were checked by the ninhydrin test described by Kaiser (Kaiser et al., 1970), or micro-cleavages performed with a microwave apparatus CEM Discover™ single-mode MW reactor (CEM Corporation, Matthews, NC, United States). Resins with peptides 2-4 were N-terminal acetylated before cleavage, using twice Ac_2_O in a DMF solution for 10 min at room temperature. Final cleavage and side-chain deprotections were performed using a mixture of TFA/TIS/H_2_O (95:2.5:2.5, v/v/v) at room temperature. After 2.5 h each resin was filtered off and the solution was concentrated flushing with N_2_. Each peptide was precipitated from cold Et_2_O, centrifuged, and lyophilized.

The crude peptides were purified by Reverse-Phase Flash Liquid Chromatography (RP-HPLC) on an Isolera One Flash Chromatography (Biotage, Uppsala, Sweden) using a SNAP Ultra C18 column (25 g) at 20 ml/min. Eluent systems: 0.1% (v/v) TFA in H_2_O (A), 0.1% (v/v) TFA in ACN (B) (elution gradient reported in Supplementary Table S1). The second step of purification of the peptides was performed by semipreparative RP-HPLC on a Waters instrument (Separation Module 2695, detector diode array 2996) using a Sepax Bio-C18 column (Sepax Technologies, Newark, United States) (5 μM, 250 mm × 10 mm), at 4 ml/min using the solvent systems A (0.1% TFA in H_2_O) and B (0.1% TFA in ACN). Characterization of the peptides was performed by analytical HPLC using a Waters ACQUITY HPLC coupled to a single quadrupole ESI-MS (Waters® ZQ Detector, Waters Milford, MA, United States) supplied with a BEH C18 column (1.7 μm, 2.1 mm × 50 mm) at 35°C, 0.6 ml/min with the solvent systems A (0.1% TFA in H_2_O) and B (0.1% TFA in ACN). Gradient elution was performed with a flow of 0.6 ml/min and started at 10% B, with a linear increase to 90% B in 5 min. The analytical data are reported in the Supplementary Material (Supplementary Table S1 and Supplementary Figures S1–S6).

### 2.3 Immunoassays

#### 2.3.1 Sample Sera Collection

Fifteen Multiple Sclerosis (MS) patients were recruited in the Multiple Sclerosis Clinical Care and Research Centre, Department of Neurosciences, Reproductive Sciences and Odontostomatology, Federico II University (Naples, Italy). The relapsing-remitting MS (RR-MS) patients were previously diagnosed after a lumbar puncture, cerebrospinal fluid analysis, and MRI examination and fulfilled the established international diagnostic criteria (Polman et al., 2011; Thompson et al., 2018). The present study was conducted in accordance with the Declaration of Helsinki. The performed experimental protocols were approved by the Ethics Committee 2006 (protocol n. 120/06) and 2017 (protocol n. 160/17). Blood samplings were performed during the routine follow-up of patients, while the healthy control samplings were carried out during routine health checks or blood donations. Sera samples were obtained for diagnostic purposes from patients and healthy controls who had given their informed consent. Blood samples were centrifuged at 4,000 rpm for 10 min and sera supernatant was stored at -20°C until use.

#### 2.3.2 Inhibition ELISA

Nunc-Immuno MicroWell 96 well polystyrene ELISA plates (NUNC Maxisorb, product code M9410, Merck, Milan, Italy) were coated with a solution 10 μg/ml of the peptide antigens in pure carbonate buffer 0.05 M (pH 9.6) adding 100 μL/well, and microplates were incubated at 4°C overnight. Wells were washed (5×) with washing buffer (0.9% NaCl, 0.05% Tween 20) using an automatic Hydroflex microplate washer (Tecan Italia, Milan, Italy). Nonspecific binding sites were blocked with 100 μL/well of fetal bovine serum (FBS) buffer solution (10% in washing buffer) at room temperature for 1 h. Antibody affinity was measured following the competitive ELISA previously reported (Real-Fernández et al., 2015). Semi-saturating sera dilution was previously calculated in preliminary titration curves (absorbance 0.7). Seven different concentrations of each synthetic antigenic peptide probe were used as inhibitors. Then, sera samples at the selected dilution were incubated in parallel with increasing concentrations of antigens (range 1 × 10^–10^ to 1 × 10^–4^ M) for 1 h at room temperature. All competitive experiments were performed in triplicate. After washes (3×), uninhibited antibodies were identified by adding 100 μL/well of alkaline phosphatase-conjugated to anti-human immunoglobulin G or M (IgG and IgM, Merck, Milano, Italy) diluted 1:3,000 (IgG) and 1:200 (IgM) in FBS buffer. The microplates were then incubated 3 h at room temperature and, after washes (3×), 100 μL of substrate solution consisting of 1 mg/ml p-nitrophenyl phosphate pNPP (Merck, Milan, Italy) and MgCl_2_ 0.01 M in carbonate buffer (pH 9.6) were added. After approximately 30 min, the reaction was stopped with 1 M NaOH solution (50 μL/well), and the absorbance was read in a multichannel ELISA reader (Tecan Sunrise, Männedorf, Switzerland) at 405 nm. Antibody titer values were calculated as (mean Abs of serum triplicate)—(mean Abs of blank triplicate) representing graphically the absorbance inhibition percentage. One positive and one negative serum, as references, were included in each plate for further normalization. Each experiment was performed at least twice in different days. Within-assays and between-assays coefficients of variations were below 10%. Calculated half maximal inhibitory concentrations (IC_50_) are reported for each antigen.

#### 2.3.3 Solid-Phase ELISA (SP-ELISA)

Immunoassays were performed to evaluate IgM or IgG antibodies in sera by SP-ELISA. At this purpose, the synthetic antigens were coated on 96-well plates (NUNC Maxisorb, product code M9410, Merck, Milan, Italy). Coating conditions were set-up independently for each peptide and results are reported in the **Supplementary Material**. Polystyrene 96-well ELISA plates were coated with 100 μL/well of a 10 μg/ml solution of synthetic peptide antigens 1-6 diluted in pure carbonate buffer 0.05 M (pH 9.6), independently. After overnight incubation at 4°C, plates were washed (3×) using washing buffer. Nonspecific binding sites were blocked with 100 μL/well of fetal bovine serum buffer (10% FBS in washing buffer) at room temperature for 1 h. FBS buffer was removed, and plates were incubated overnight at 4°C with sera (diluted 1:100 in FBS buffer, 100 μL/well). After three washes, plates were treated with 100 μL/well of anti-human IgG or IgM alkaline phosphatase-conjugated specific antibodies diluted in FBS buffer 1:3,000 (IgG) and 1:200 (IgM) for all tested antigens. After 3 h of incubation at room temperature and washes (3×), 100 μL of substrate buffer (1 mg/ml *p*NPP, MgCl_2_ 0.01 M in carbonate buffer, pH 9.6) was added to each well. Colorimetric reaction was carried out adding 100 μl of substrate reaction solution (1 mg/ml *p*NPP, MgCl_2_ 0.01 M in carbonate buffer, pH 9.6) to each well and plates were read at 405 nm using a TECAN plate reader. After 30 min, the reaction was stopped with 1 M NaOH solution (50 μL/well) and the absorbance was read in a multichannel ELISA reader (Tecan Sunrise, Männedorf, Switzerland) at 405 nm. Antibody titer values were calculated as (mean Abs of serum triplicate)—(mean Abs of blank triplicate) representing graphically the calculated mean values. One positive and one negative serum, as references, were included in each plate for further normalization. Each experiment was performed at least twice in different days. Within-assays and between-assays coefficients of variations were below 10%.

### 2.4 Circular Dichroism

CD spectra were recorded on JASCO J-815 with increasing temperature from 10 to 60°C in increments of 5°C between *λ* = 270 and 185 nm. The CD measurements were carried out in H_2_O, a mixture of H_2_O:TFE (50:50, v:v) and PBS (phosphate buffered saline, pH 7.4, containing: sodium chloride, potassium chloride, sodium phosphate dibasic, potassium phosphate monobasic). The spectra were registered with the following parameters: 0.2 nm resolution, 1.0 nm bandwidth, 20 mdeg sensitivity, 0.25 s response, 100 nm min^−1^ scanning speed, 5 scans, and 0.02 cm cuvette path length. The CD spectra of solvents were recorded and subtracted from the raw data. The spectra were corrected by a baseline that was measured with the identical solvent in the same cell. The CD intensity is given as mean residue molar ellipticity (θ) [deg × cm^2^ × dmol^−1^].

### 2.5 NMR

The samples for NMR spectroscopy were prepared by dissolving the appropriate amount of peptide in 50 mM potassium phosphate buffer (pH 6.5), 10% D_2_O, and 100 mM DPC-d_38_. NMR spectra were recorded on a Varian INOVA 600 MHz spectrometer equipped with a z-gradient 5 mm triple-resonance probe head. Spectra were recorded at a temperature of 25°C. The spectra were calibrated relative to TSP (0.00 ppm) as internal standard. One-dimensional (1D) NMR spectra were recorded in the Fourier mode with quadrature detection. The water signal was suppressed by gradient echo (Hwang and Shaka, 1995). 2D DQF-COSY (Piantini et al., 1982; Marion and Wüthrich, 1983), TOCSY (Braunschweiler and Ernst, 1983), and NOESY (Jenner et al., 1979) spectra were recorded in the phase-sensitive mode. Data block sizes were 2048 addresses in T2 and 512 equidistant T1 values. Before Fourier transformation, the time domain data matrices were multiplied by shifted sin2 functions in both dimensions. A mixing time of 70 ms was used for the TOCSY experiments. NOESY experiments were run with mixing time of 100 ms.

## 3 Results and Discussion

### 3.1 Design of the Synthetic Myelin Basic Protein Peptides

Many publications indicate the presence of an immunodominant sequence in a specific region of MBP. One report described the sequence MBP (83–101) as a potential immunodominant T-cell epitope restricted to HLA-DR2b (DRB1*15:01) in MS (Ota et al., 1990), while others reported that MBP (89–101) peptide has two registers for binding to DR2a and DR2b (Kim et al., 2014). Other authors identified MBP (87–106) (Martin et al., 1990), MBP (83–101) (Hansen et al., 2007), and other overlapping sequences as T-cell epitopes inside the MBP protein. Importantly, these MBP regions match with B-cell epitopes previously described (Lolli et al., 2005; Warren et al., 2006; Mameli et al., 2014).

With the idea in mind to cover the whole immunodominant region of MBP, we synthesized and tested the peptide MBP (81–106) (1). Moreover, considering that the sensitivity of the peptide-based ELISA might be limited when short antigen sequences are used, we designed the elongated sequence MBP (76–116) (2), in order to achieve better antigen exposition in the solid-phase conditions of the ELISA (Nuti et al., 2020). With the aim of clarifying the amino-acid residues specifically involved in the B-cell epitope recognition, we divided the whole sequence of MBP (76–116) (2) into two fragments: MBP (76–96) (3) and MBP (97–116) (4). Amino- and carboxy-termini of peptides were acetylated and amidated, respectively, in order to remove free terminal charges, which are not present in the native protein sequence and may interfere with antibody recognition (Van Regenmortel, 2001). Finally, we also synthesized the shorter sequences MBP (81–92) (5) and MBP (99–106) (6), to be tested in competitive ELISA.

### 3.2 Biological Activity

#### 3.2.1 Solid-phase ELISA

**FIGURE 1:**
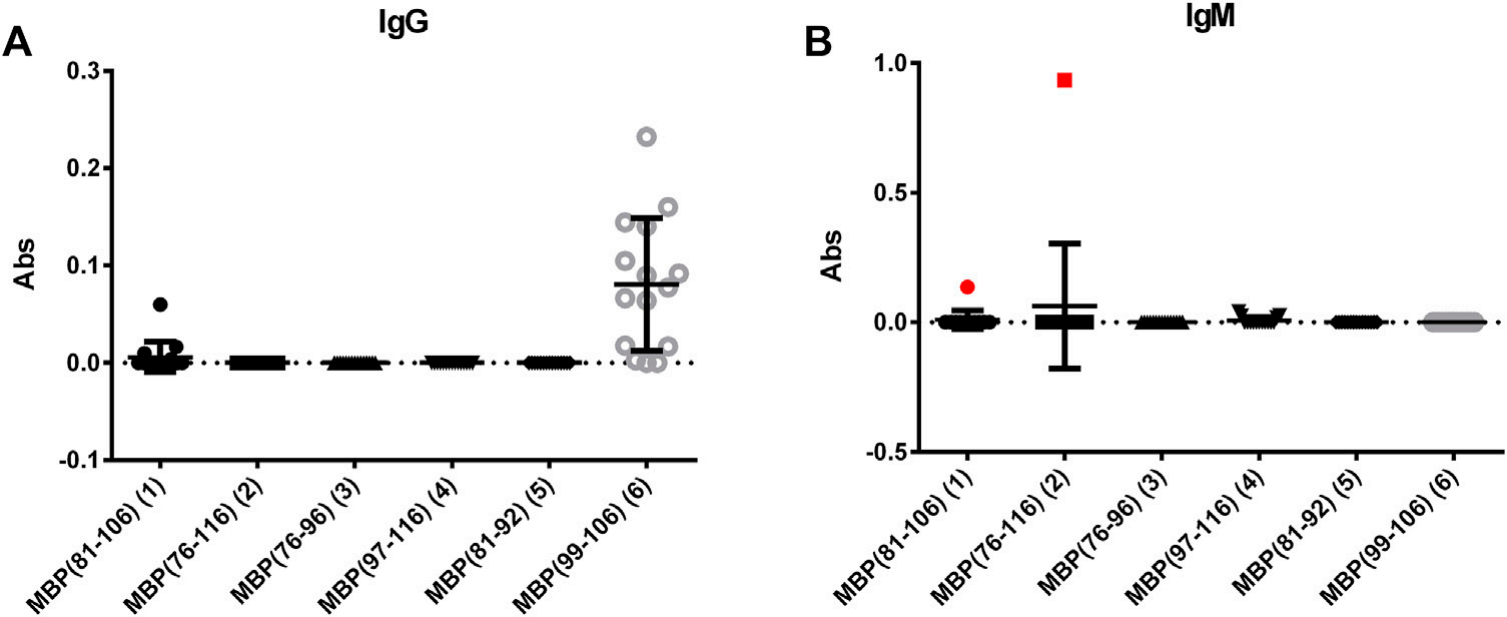
Mean antibody titers to MBP peptide antigens for IgGs **(A)** and IgMs **(B)** of MS patient sera. IgM antibody responses of the MS serum to the coated peptides MBP (81–106) (1) and MBP (76–106) (2) are plotted in red.

Fifteen sera from MS patients were screened using the synthetic peptides 1–6 as antigens in solid-phase ELISA. To this purpose, preliminary tests were performed to set-up the optimal conditions for the coating of the different peptide antigens onto the ELISA plates. Accordingly, we evaluated IgG and IgM antibody titres to each relevant peptide (Figure 1), observing a nonspecific IgG antibody reactivity against peptide MBP (99–106) (6). To a lesser extent, also against peptide MBP (81–106) (1). A deeper analysis of these data indicated that peptide 6 is too short to be efficiently coated and/or exposed on the ELISA plate, favouring the nonspecific signals observed (Supplementary Figure S13). At variance, peptides 2-5 did not detect IgG-type antibodies. On the other hand, IgM antibodies were detected in one representative patient serum with peptide MBP (76–116) (2) and, to a lesser way, with peptide MBP (81–106) (1) [1 out of 15 patients (7%)] (Figure 1B). Interestingly, whereas the intensity of IgMs signal is clear for peptide MBP (76–116) (2), the peptide MBP (81–106) (1) slightly recognized IgGs and IgMs. This finding can be explained by the longer peptide MBP (76–116) (2), which may contain an extended epitope, or features an optimal exposition on the ELISA plate, not obtained by the shorter peptide MBP (81–106) (1), but important to capture pentameric IgM antibodies. This interesting result let us to hypothesize that the structures of peptide 1 may reproduce the correct antigen presentation, i.e., the one observed in the whole protein, thus enabling optimal antibody recognition of IgM antibodies. In fact, we already reported that, generally speaking, IgG antibodies can be more easily identified in ELISA using synthetic peptides as antigens, as compared to IgMs, possibly due to the pentameric spatial orientation of the latter. Previously, we overcome this problem using multivalent peptides to exploit IgMs higher avidity (Nuti et al., 2020; Mazzoleni et al., 2021). Interestingly, we herein observed that the monomeric, linear peptide MBP (76–116) (2) is able to detect a high, stable, and reproducible IgM antibody titer.

An additional explanation for the low reactivity generally observed for both IgGs and IgMs with most of the tested samples stems from the observation that a high percentage of patients are under immunosuppressive treatment, and they are not in the initial stage of the disease. In fact, it has been previously reported that anti-MBP IgM antibodies decrease in patients with a longer disease duration (Egg et al., 2001). Moreover, it should be noted that the recognized representative patient is not following any immunosuppressive treatment.

#### 3.2.2 Inhibition ELISA

**FIGURE 2:**
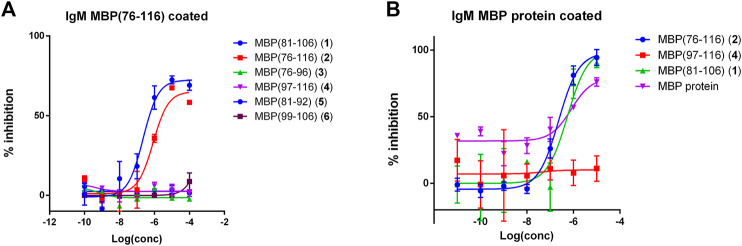
Competitive ELISA obtained coating the peptide antigen MBP (76–116) (2) **(A)** or the MBP protein **(B)**. Inhibition curve of IgMs using peptides and protein as inhibitors at different concentrations. Results show the inhibition activity % (ordinate axis) of the reference MS serum for IgMs vs. antigen concentrations on a logarithmic scale (abscissas axis). Antibody titer values were calculated as (mean Abs of serum triplicate)—(mean Abs of blank triplicate) representing graphically the calculated mean values ± the standard deviation.

We focused our attention on the IgM antibody response to peptide MBP (76–116) (2), with the aim of verifying the specificity of the observed signals in a competitive solid-phase ELISA. To this purpose, MBP (76–116) (2) was coated on the plate and all the synthesized MBP sequences, including MBP (76–116) (2) itself, were individually tested at different concentrations as inhibitors of IgM antibody binding in the representative serum. The results showed in Figure 2A indicate that peptides MBP (81–106) (1) and MBP (76–116) (2) were able to inhibit IgM antibody binding to peptide 2 in the tested MS serum, showing an IC_50_ of 2.2∙10^–7^ and 8.4∙10^–7^, respectively (Table 2). These results confirm that IgM antibody response to peptide MBP (76–116) (2) is concentration-dependent and assess the specificity of the IgM recognition, previously observed in SP-ELISA. Moreover, we performed the inhibition experiments coating the MBP protein and using peptides MBP (81–106) (1), MBP (76–116) (2), MBP (97–116) (4), and the MBP protein as inhibitors (Figure 2B). Results showed the cross-reactivity between peptides MBP (81–106) (1) and MBP (76–116) (2) and the whole MBP protein, and peptides were able to inhibit IgM antibody binding to MBP protein in the tested MS serum with comparable IC_50_ (Table 2). The greater affinity observed in the competitive experiment with peptide MBP (81–106) (1) indicates that, while the elongation of the sequence featured by peptide MBP (76–116) (2) is fundamental for antibody recognition in solid-phase ELISA, the shortened sequence MBP (81–106) (1) is recognized more efficiently by antibodies in the solution conditions of the competitive experiments. In light of these results, we decided to study the secondary structure of peptides MBP (81–106) (1) and MBP (76–116) (2) by circular dichroism.

**TABLE 1:** Synthesized MBP peptides.

Peptide	Fragment	Sequence
**1**	MBP (81–106)	-----TQDENPVVHFFKNIVTPRTPPPSQGK----------
**2**	MBP (76–116)^a^	SQHGRTQDENPVVHFFKNIVTPRTPPPSQGKGRGLSLSRFS
**3**	MBP (76–96)^a^	SQHGRTQDENPVVHFFKNIVT--------------------
**4**	MBP (97–116)^a^	---------------------PRTPPPSQGKGRGLSLSRFS
**5**	MBP (81–92)	-----TQDENPVVHFFK------------------------
**6**	MBP (99–106)	-----------------------TPPPSQGK----------

a N-terminal acetylated and C-terminal amide.

**TABLE 2:** Calculated IC_50_ values of anti-MBP (76–116) or anti-MBP protein IgM antibodies of MS serum to MBP (76–116) (2) and MBP (81–106) (1). Values are reported as 95% confidence interval for the calculated mean IC_50_ ± the standard error (SEM).

Coated antigen	Inhibitor	IC_50_ (IgM)
MBP (76–116) (2)	MBP (81–106) (1)	(2.2 ± 0.18)∙10^–7^
	MBP (76–116) (2)	(8.4 ± 0.24)∙10^–7^
MBP protein	MBP (81–106) (1)	(5.5 ± 0.31)∙10^–7^
	MBP (76–116) (2)	(2.3 ± 0.07)∙10^–7^
	MBP protein	(7.9 ± 0.34)∙10^–7^

### 3.3 Circular Dichroism

**FIGURE 3:**
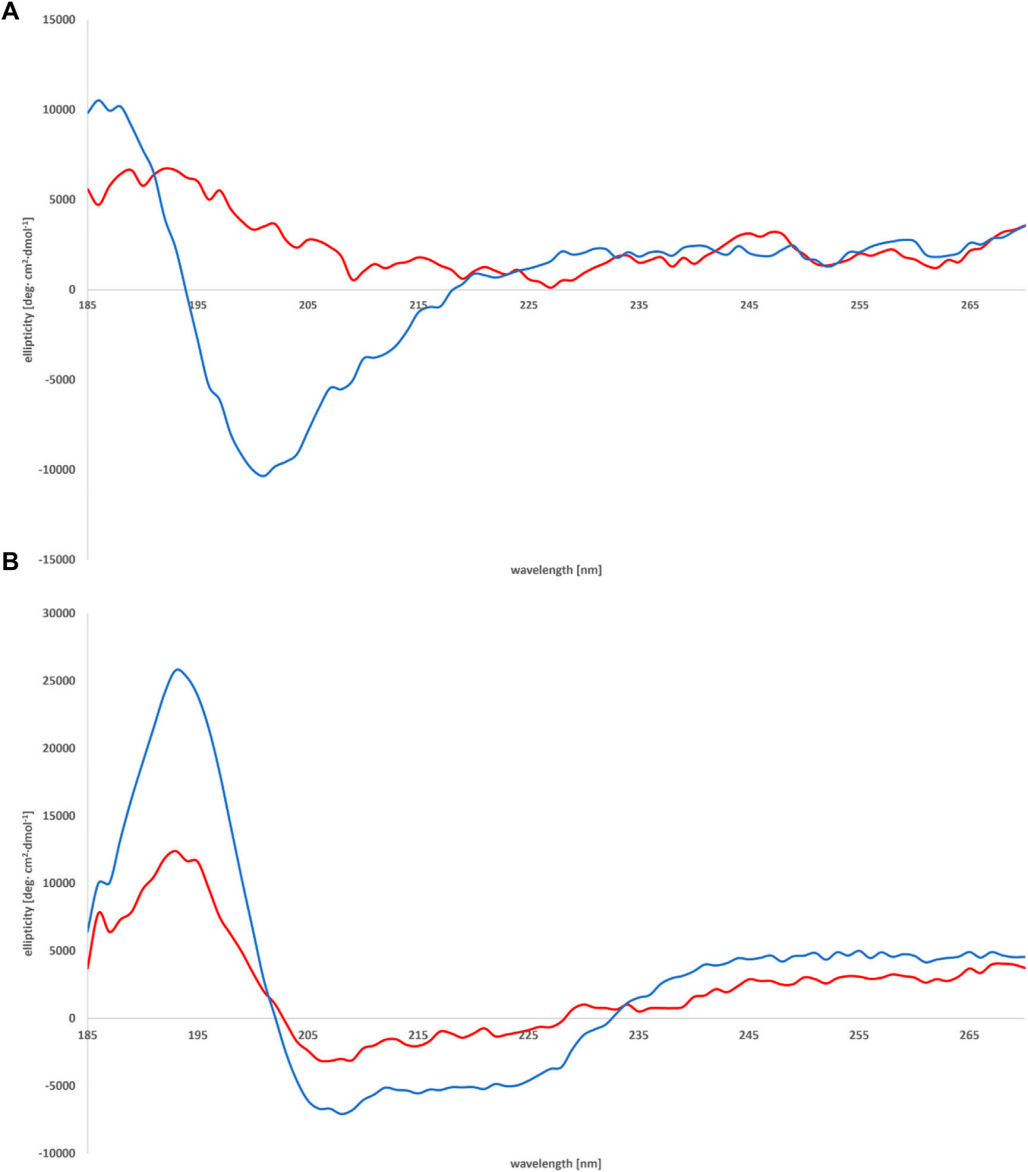
CD spectra of peptides MBP (81–106) (1) (blue line) and MBP (76–116) (2) (red line) measured in water **(A)** and mixture of H_2_O:TFE (50:50, v:v) **(B)** at 25°C.

A preliminary screening of the conformational preferences of peptides MBP (81–106) (1) and MBP (76–116) (2) indicate that these peptides appear randomly structured in water solution (Figure 3A; Supplementary Figure S14). We subsequently studied the conformational behavior of these peptides in H_2_O:TFE (50:50, v:v), a solvent mixture known to act as stabilizing agent (Campagna et al., 1998). Moreover, the use of fluoroalcohol provides additional stability by removing the water molecules from the surroundings of the peptides (Khandelwal et al., 1999). The spectra obtained in the solvent mixture displayed two negative bands at 222 and 208 nm (n→π* and π→π* transitions) and one positive band at 190 nm (π→π* transition) (Figure 3B), characteristic for helical structures according to the literature (Bradley et al., 1990; Bates et al., 2004; Ahmed et al., 2012; Miles et al., 2022). Indeed, predictions based on amino acid composition of these peptides indicate that they show a tendency to form helical structures (Yang et al., 1997; Bates et al., 2004; Ahmed et al., 2012). Moreover, it may be assumed that there were no spectral differences of secondary peptide structure at various temperatures (Supplementary Figure S14, S15). Interestingly, the shorter peptide MBP (81–106) (1) displays higher tendency to adopt an helical conformation, as compared to the longer analogue MBP (76–116) (2) (% helix 14 *vs*. 3) (Figure 3B) (Sommese et al., 2010). Apparently, the elongation at N- and C-termini of the peptide causes the disability in forming the helix. Furthermore, a different agent stabilizing the helix motif in the structure of MBP (81–106) (1) is the presence at the C- and N-termini of positively and negatively charged amino acids able to form salt bridges (Forood et al., 1993). At variance, the longer peptide MBP (76–116) (2) includes many positively charged residues, i.e., arginine residues that produce a destabilized effect on helix conformation by electrostatic repulsion (Sitkoff et al., 1994).

**FIGURE 4:**
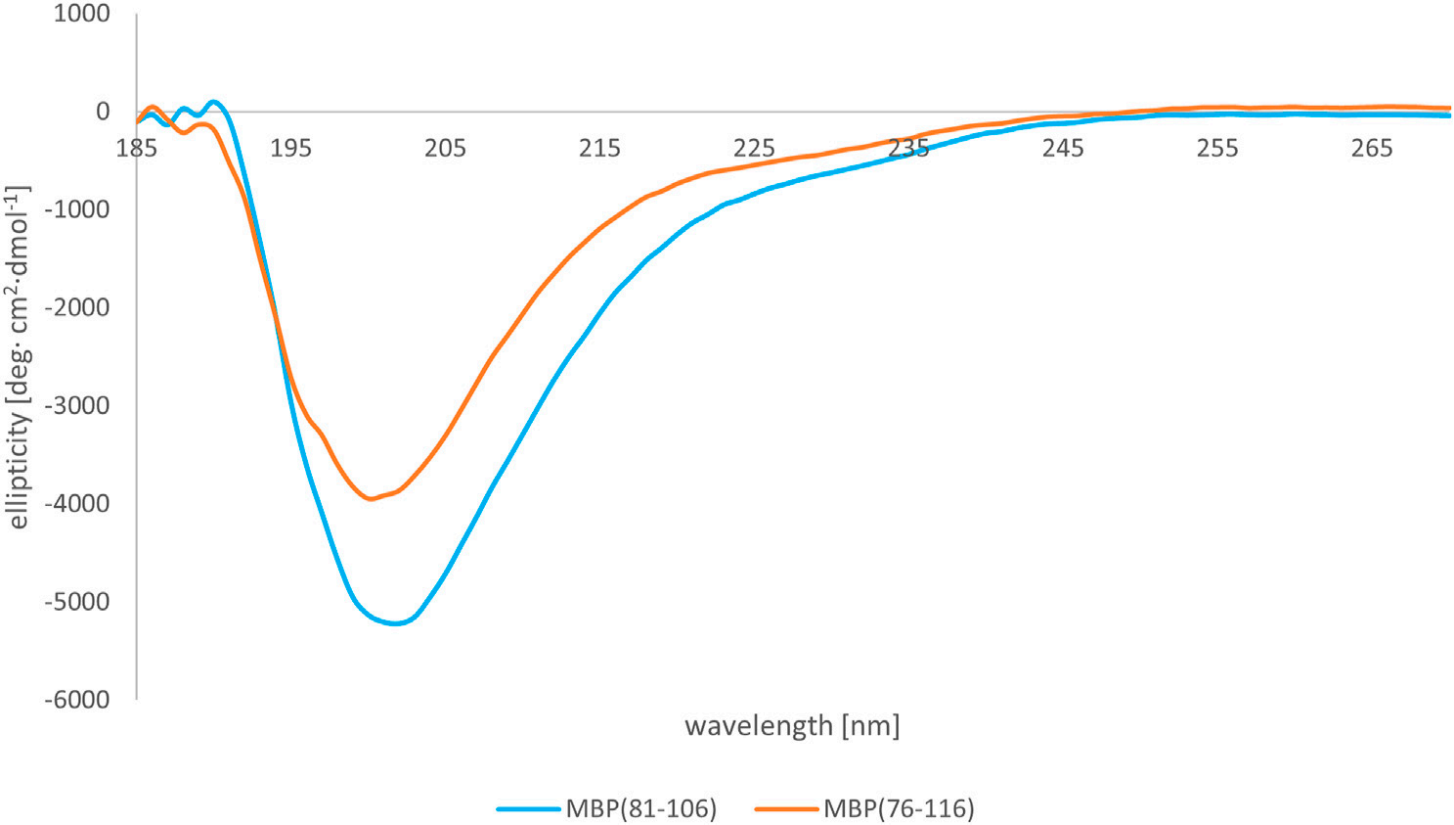
CD spectra of peptides MBP (81–106) (1) (blue line) and MBP (76–116) (2) (red line) in PBS at 25°C.

**FIGURE 5:**
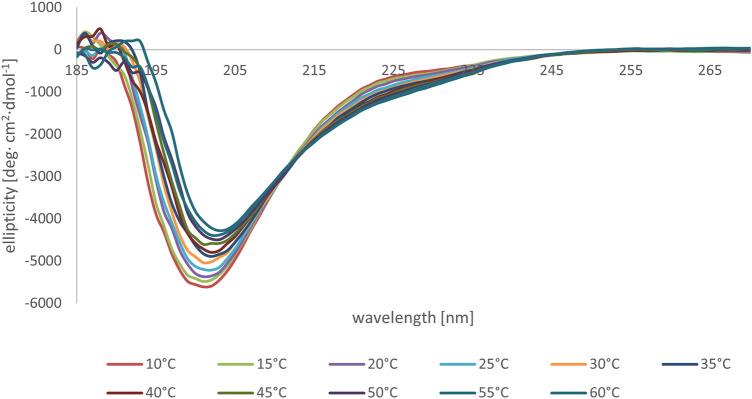
CD spectra of peptide MBP (81–106) (1) in PBS at various temperatures.

**FIGURE 6:**
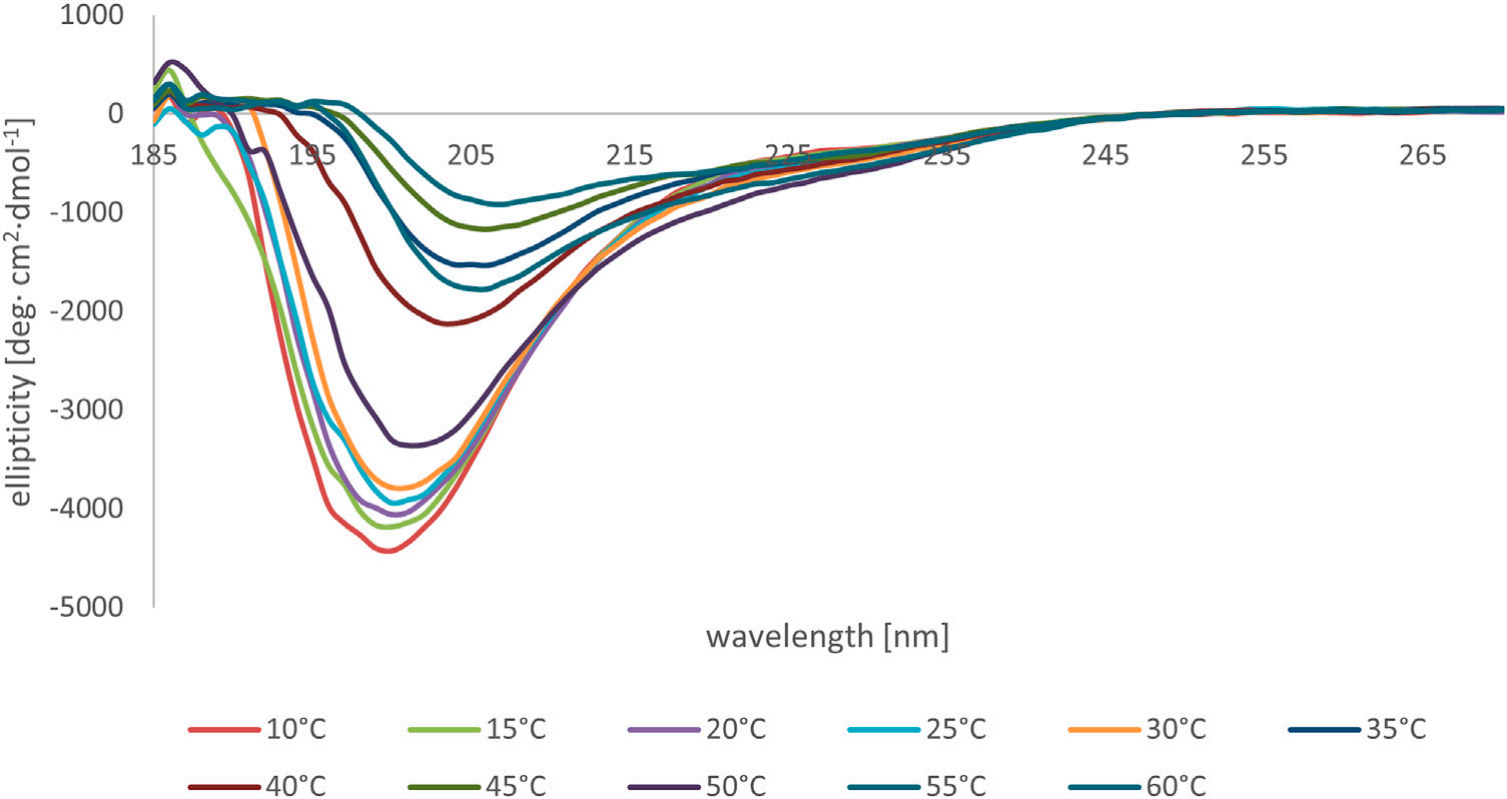
CD spectra of peptide MBP (76–116) (2) in PBS at various temperatures.

We subsequently studied the conformational preferences of peptides MBP(81–106) (1) and MBP (76–116) (2) in PBS, which is the most frequently used solvent to maintain physiological pH (White, 2002). These data indicate that the peptides present random coil structures (Figure 4). In fact, the obtained spectra displayed a single band with negative ellipticity at approximately 200 nm characteristic for unordered peptides. However, peptide MBP (81–106) (1) presents a more ordered structure, as can be inferred by the small but significant shift at higher wavelength of the minimum of MBP (81–106) (1) compared to MBP (76–116) (2) (Muruganandam et al., 2013; Lanthier et al., 2014). Moreover, we can assume there were no differences of secondary structure at various temperatures for MBP(81–106) (1) (Figure 5). In contrast, increasing the temperature of MBP (76–116) (2) led to a shift of the minimum to lower wavelength and a strong decrease of the signal intensity (Figure 6). This result can derive from a temperature-induced peptide self-aggregation, likely in beta structures (minimum at about 205 nm). The partial aggregation can account for the reduced ability of MBP (76–116) (2) to interact with the IgM antibody.

### 3.4 NMR

Conformational behavior of peptides MBP (81–106) (1) and MBP (76–116) (2) was studied in DPC micelle (100 mm) by solution NMR spectroscopy. Almost complete ^1^H NMR assignments were not was obtained for peptide 1 while some residues of 2 could not be assigned due to strong signals overlapping (Supplementary Tables S2, S3). From chemical shift and NOE interaction patterns (Supplementary Tables S2, S3; Supplementary Figure S16), the two peptides have stable helical structure along residues 87–96, while the remaining regions did not show diagnostic parameters of any secondary structure. They are henceforth in random coil conformation. These results are in agreement with those reported in the literature (Ahmed et al., 2012) about a 36-residue peptide fragment of murine MBP corresponding to residues 76–111. Comparing the chemical shift values peptides MBP (81–106) (1) and MBP (76–116) (2), which are not significantly different, the helical structure turns out to have the same stability regardless of the sequence length. Superposition of chosen regions of the NOESY spectra of the two peptides (Supplementary Figure S16) gives a visual confirmation of this result.

## 4 Conclusion

In MS the myelin sheath is severely damaged by the autoimmune response, including autoreactive antibodies, therefore the characterization of antigens, particularly those expressed by proteins in the CNS, is highly relevant. In this study, we investigated the MBP protein, an important constituent of the myelin membrane. New insights in the anti-MBP antibodies and their pathogenicity can contribute to clarify their role, debated in the literature for many years. To this aim, we synthesized peptides covering the MBP (76–116) (2) fragment, including the immunodominant B-cell epitope MBP (84–104), and tested them both in solid-phase and competitive ELISA with MS patient sera. We focused our attention on IgM antibodies, observed in one representative serum using the peptides MBP (81–106) (1) and MBP (76–116) (2), while the other tested sequences showed in general low reactivity, for both IgGs and IgMs. The reactivity of peptides 1 and 2 is particularly relevant since frequency of serum anti-MBP IgMs is lower in patients with a long-term disease duration. Competitive ELISA experiments confirmed the specificity of the interaction and perhaps high antibody affinity to these peptide sequences.

NMR conformational analysis in DPC indicates that the two peptides MBP (81–106) (1) and MBP (76–116) (2) display a stable helical conformation along residues 87–96, while the remaining regions display unordered conformation. CD experiments in H_2_O:TFE mixture showed that the shorter peptide MBP (81–106) (1) displays a slightly higher tendency to adopt an helical conformation, which may be considered the bioactive conformation of the epitope recognized by IgMs, since the competitive ELISA experiments show a 4-fold greater affinity of IgMs for this peptide, as compared to the longer analogue. However, the longer peptide MBP (76–116) (2) appears to be much more suitable to be efficiently coated on the plate, correctly exposing the helical epitope to capture IgMs in solid-phase. At variance, the correct epitope exposition is hampered by the flanking flexible N- and C-terminal regions, when the longer peptide is tested in solution.

In conclusion, the efficacy of the experimental approach based on the use of designed peptide sequences as synthetic antigenic probes to characterize antibodies in patient sera is greatly enhanced by the optimisation of the procedure to synthesize specifically modified and unique molecules (Nuti et al., 2010). Moreover, we demonstrated the possible positive role of chain elongation in solid-phase ELISA, enabling the detection of antibodies that cannot be identified by shorter sequences, even if they contain an epitope. These facts explain how minor changes in the MBP sequence and structure may contribute to the controversial results reported about its antibody reactivity. The selection of the methodology with the correct and well-exposed structures, is fundamental to improve antibody identification. Further studies based on a larger cohort of recently diagnosed and untreated MS patients could contribute to clarify the role of MBP in Multiple Sclerosis.

## Data Availability

The raw data supporting the conclusions of this article will be made available by the authors, without undue reservation.
