# Supplementary material for: Role of Helical Structure in MBP Immunodominant Peptides for Efficient IgM Antibody Recognition in Multiple Sclerosis
Source: Front Chem. 2022 Jun 20;10:885180. doi: 10.3389/fchem.2022.885180 (PMC9250970; doi:10.3389/fchem.2022.885180)
Supplement: Supplementary file 1 [file DataSheet1.pdf]

Supplementary material

Role of helical structure in MBP immunodominant peptides for efficient IgM antibody recognition in Multiple Sclerosis

Agnieszka Staśkiewicz[1,2], Michael Quagliata[1], Feliciana Real-Fernandez[1], Francesca Nuti[1], Roberta Lanzillo[3], Vincenzo Brescia-Morra[3], Hendrik Rusche[4,5], Michal Jewginski[2], Alfonso Carotenuto[6], Diego Brancaccio[6], Rina Aharoni[7], Ruth Arnon[7], Paolo Rovero,[8] Rafal Latajka[2], and Anna Maria Papini[1,5*]

[1]Interdepartmental Research Unit of Peptide and Protein Chemistry and Biology, Department of Chemistry "Ugo Schiff", University of Florence, Sesto Fiorentino, Italy

[2]Department of Bioorganic Chemistry, Faculty of Chemistry, Wroclaw University of Science and Technology, Wroclaw, Poland

[3]Multiple Sclerosis Clinical Care and Research Centre, Department of Neurosciences, Reproductive Sciences and Odontostomatology, Federico II University, Naples, Italy

[4]Fischer analytics GmbH, Weiler, Germany

[5]CY PeptLab Platform of Peptide and Protein Chemistry and Biology and UMR 8076 CNRS-BioCIS, CNRS, CY Cergy Paris Université, Neuville sur Oise, France

[6]Department of Pharmacy, University of Naples "Federico II", Naples, Italy

[7]Department of Immunology, The Weizmann Institute of Science, Rehovot, Israel

[8]Interdepartmental Research Unit of Peptide and Protein Chemistry and Biology, Department of NeuroFarBa, University of Florence, Sesto Fiorentino, Italy

***Correspondence:**
Prof. Anna Maria Papini, PhD
annamaria.papini@unifi.it

Keywords: multiple sclerosis, circular dichroism, immune response, synthetic helical peptides, myelin basic protein, NMR, peptide-antigen based ELISA.

Analytical characterization of peptides

The crude synthetic peptides were purified by Reverse-Phase Flash Liquid Chromatography (RP-FLC) on an Isolera One Flash Chromatography (Biotage, Uppsala, Sweden) using a SNAP Ultra C18 column (25 g) at 20 mL/min as solvent systems H_2O (MilliQ) and ACN (gradient reported in **Table S1**. The second step of purification of the peptides was performed by semipreparative RP-HPLC on a Waters instrument (Separation Module 2695, detector diode array 2996) using a Sepax Bio-C18 column (Sepax Technologies, Newark, USA) (5 µm, 250 × 10 mm), at 4 mL/min with solvent systems A (0.1% TFA in H_2O) and B (0.1% TFA in ACN). Characterization of the peptides was performed by analytical HPLC using a Waters ACQUITY HPLC coupled to a single quadrupole ESI-MS (Waters® ZQ Detector, Waters Milford, MA, USA) supplied with a BEH C18 (1.7 µm 2.1 × 50 mm) column at 35 °C, at 0.6 mL/min with solvent systems A (0.1% TFA in H_2O) and B (0.1% TFA in ACN). Gradient elution was performed with a flow of 0.6 mL/min and started at 10% B, with a linear increase to 90% B in 5 min.

Table S1. Analytical characterization of peptides

Peptide	*Fragment*	*Purification gradient method (%)*	*HPLC gradient (% B)[a] R_f (min)*	*HPLC purity (%)*	*ESI-MS (m/z) found[c] (calcd)*
**1**	MBP (81-106)	0-60 in 25 min	5-95<br>3.90 min	>95	979.4 (979.1)[c]
**2**	MBP (76-116)*	20-60 in 25 min	5-60<br>3.88 min	>95	1151.9 (1151.5)[d]
**3**	MBP (76-96)*	10-60 in 25 min	5-95<br>4.10 min	>95	832.7 (832.6)[c]
**4**	MBP (97-116)*	20-60 in 25 min	5-95<br>3.32 min	>95	723.3 (723.2)[c]
**5**	MBP (81-92)	0-60 in 25 min	5-95<br>3.63min	>95	731.4 (731.3)[e]
**6**	MBP (99-106)	0-10 in 10 min	1-20[b]<br>1.55 min	>95	811.6 (811.9)[f]

The peptides were characterized by RP-HPLC Alliance Chromatography system (Waters, Milford Massachusetts, USA) with a BEH C18 (1.7 µm 2.1× 50 mm) column at 35°C, 0.6 mL/min, coupled to a single quadrupole ESI-MS Micromass ZQ (Waters, Milford Massachusetts, USA). Eluents: 0.1% (v/v) TFA in H_2O (A) and 0.1% (v/v) TFA in AcCN (B), λ 215 nm. Gradient times: [a]10min; [b]5min. ESI-MS: detected as [c]$[M+3H]^{3+}$, [d]$[M+4H]^{4+}$, [e]$[M+2H]^{2+}$; [f]$[M+H]^{+}$.

*Peptide sequence N-terminal acetylated and C-terminal amide

Table S2. ^{1}H NMR resonance assignments [a] of peptide MBP (81-106) (**1**) in 100 mM DPC solution

Residue	NH	C$^{\alpha}$H	C$^{\beta}$H	Others
Thr 81		3.87	4.19	1.29(γ)
Gln 82	8.44	4.36	2.10, 1.98	2.37(γ)
Asp 83	8.44	4.55	2.65, 2.55	
Glu 84	8.19	4.33	1.98, 1.92	2.26(γ)
Asn 85	8.99	4.91	3.01, 2.76	8.49, 6.59(δ)
Pro 86		4.43	2.50, 1.91	2.18, 2.06(γ); 3.99, 3.82(δ)
Val 87	8.34	3.78	2.33	1.07(γ)
Val 88	7.59	3.65	2.27	1.11, 1.04(γ)
His 89	8.45	4.11	3.25, 3.18	7.18(δ); 8.28(ε)
Phe 90	7.95	4.17	3.27, 3.18	6.80(δ); 7.02(ε); 7.06(z)
Phe 91	8.05	4.10	3.14, 3.07	7.36(δ); 7.23(ε); 7.17(z)
Lys 92	8.56	3.89	1.80	1.36(γ); 1.66(δ); 2.84(ε)
Asn 93	7.64	4.46	2.65, 2.54	7.48, 6.71(δ)
Ile 94	7.37	3.89	1.80	1.37, 1.02, 0.79, 0.61(γ); 0.62(δ)
Val 95	7.37	4.12	2.18	0.90(γ)
Thr 96	7.74	4.51	4.13	1.22(γ)
Pro 97		4.43	1.93, 2.30	2.04(γ); 3.77, 3.64(δ)
Arg 98	8.52	4.35	1.84, 1.76	1.66(γ); 3.19(δ); 7.32(ε)
Thr 99	8.22	4.55	4.10	1.24(γ)
Pro 100		4.68	2.33, 1.87	2.04, 1.99(γ); 3.87, 3.68(δ)
Pro 101		4.71	2.35, 1.91	2.03(γ); 3.82, 3.61(δ)
Pro 102		4.44	1.93, 2.30	2.03(γ); 3.81, 3.65(δ)
Ser 103	8.37	4.40	3.89, 3.84	
Gln 104	8.34	n.a.	2.06, 1.91	
Gly 105	8.43	3.93		
Lys 106	7.83	4.17	1.82, 1.69	1.36(γ); 2.99(ε)

[a] Obtained at 25°C, pH = 6.5, with TSP (δ 0.00 ppm) as reference shift. Chemical shifts are accurate to ±0.02 ppm. n.a. not assigned.

Table S3. ^{1}H NMR resonance assignments[a] of peptide MBP (76-116) (**2**) in 100 mM DPC solution.

Residue	NH	C$^{\alpha}$H	C$^{\beta}$H	Others
Ser 76		4.26	3.84	
Gln 77	8.58	4.31	2.04, 1.91	2.30, 2.26(γ)
His 78	8.34	4.65	3.22, 3.09	7.14(δ); 8.17(ε)
Gly 79	n.a.			
Arg 80	8.39	4.32	1.88, 1.77	1.65(γ); 3.19(δ)
Thr 81	8.33	4.31	4.22	1.20(γ)
Gln 82	8.49	4.31	2.08, 1.97	2.32(γ)
Asp 83	8.30	4.56	2.66, 2.59	
Glu 84	8.17	4.32	1.98, 1.92	2.26(γ)
Asn 85	8.99	4.91	3.01, 2.76	8.46, 6.62(δ)
Pro 86		4.43	2.50, 1.91	2.18, 2.06(γ); 3.99, 3.82(δ)
Val 87	8.32	3.78	2.33	1.07(γ)
Val 88	7.59	3.65	2.27	1.11, 1.04(γ)
His 89	8.44	4.11	3.25, 3.18	7.16(δ); 8.23(ε)
Phe 90	7.99	4.16	3.27, 3.18	6.80(δ); 7.02(ε); 7.06(z)
Phe 91	8.07	4.10	3.14, 3.07	7.36(δ); 7.23(ε); 7.17(z)
Lys 92	8.56	3.90	1.80	1.36(γ); 1.66(δ); 2.84(ε)
Asn 93	7.64	4.47	2.65, 2.55	7.47, 6.72(δ)
Ile 94	7.38	3.90	1.79	1.37, 1.02, 0.78(γ); 0.62(δ)
Val 95	7.41	4.11	2.18	0.90(γ)
Thr 96	7.77	4.50	4.13	1.21(γ)
Pro 97		4.44	1.93, 2.30	2.04(γ); 3.77, 3.62(δ)
Arg 98	8.46	4.34	1.86, 1.76	1.64(γ); 3.18(δ)
Thr 99	8.12	4.55	4.10	1.23(γ)
Pro 100		4.67	2.32, 1.87	2.03, 1.98(γ); 3.86, 3.68(δ)
Pro 101		4.70	2.35, 1.91	2.03(γ); 3.81, 3.61(δ)
Pro 102		4.44	1.94, 2.29	2.04(γ); 3.80, 3.64(δ)
Ser 103	8.35	4.39	3.83, 3.79	
Gln 104	8.43	4.36	2.15, 1.98	2.37(γ)
Gly 105	8.43	3.95		
Lys 106	8.29	4.33	1.83, 1.70	1.44(γ); 2.99(ε)
Gly 107	n.a.			
Arg 108	8.30	4.44	1.87, 1.77	1.64(γ); 3.19(δ); 7.32(ε)
Gly 109	n.a.			
Leu 110	8.18	4.35		0.90(δ)
Ser 111	8.04	4.57	3.94, 3.81	
Leu 112	8.80	4.38	1.75	0.92(δ)
Ser 113	8.22	4.45	3.84	
Arg 114	7.98	4.18	1.85, 1.72	1.60(γ); 3.19(δ)
Phe 115	7.88	4.61	3.31, 2.97	7.31(δ)
Ser 116	7.86	4.36	3.91, 3.82	

[a] Obtained at 25°C, pH = 6.5, with TSP (δ 0.00 ppm) as reference shift. Chemical shifts are accurate to ±0.02 ppm. n.a. not assigned. N-terminal CH_3CO: 2.06 ppm.

MBP(81-106) (1)

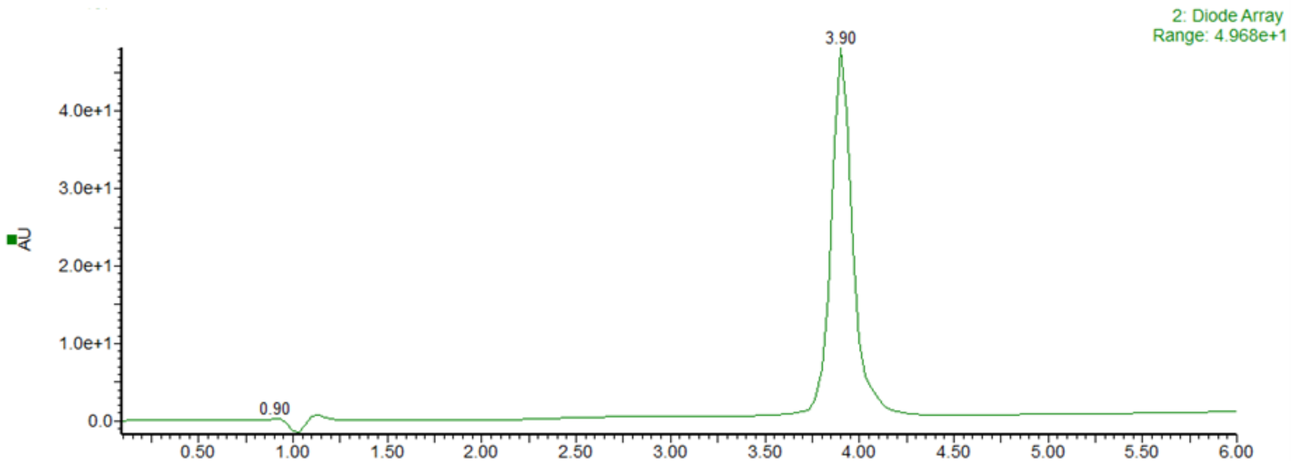

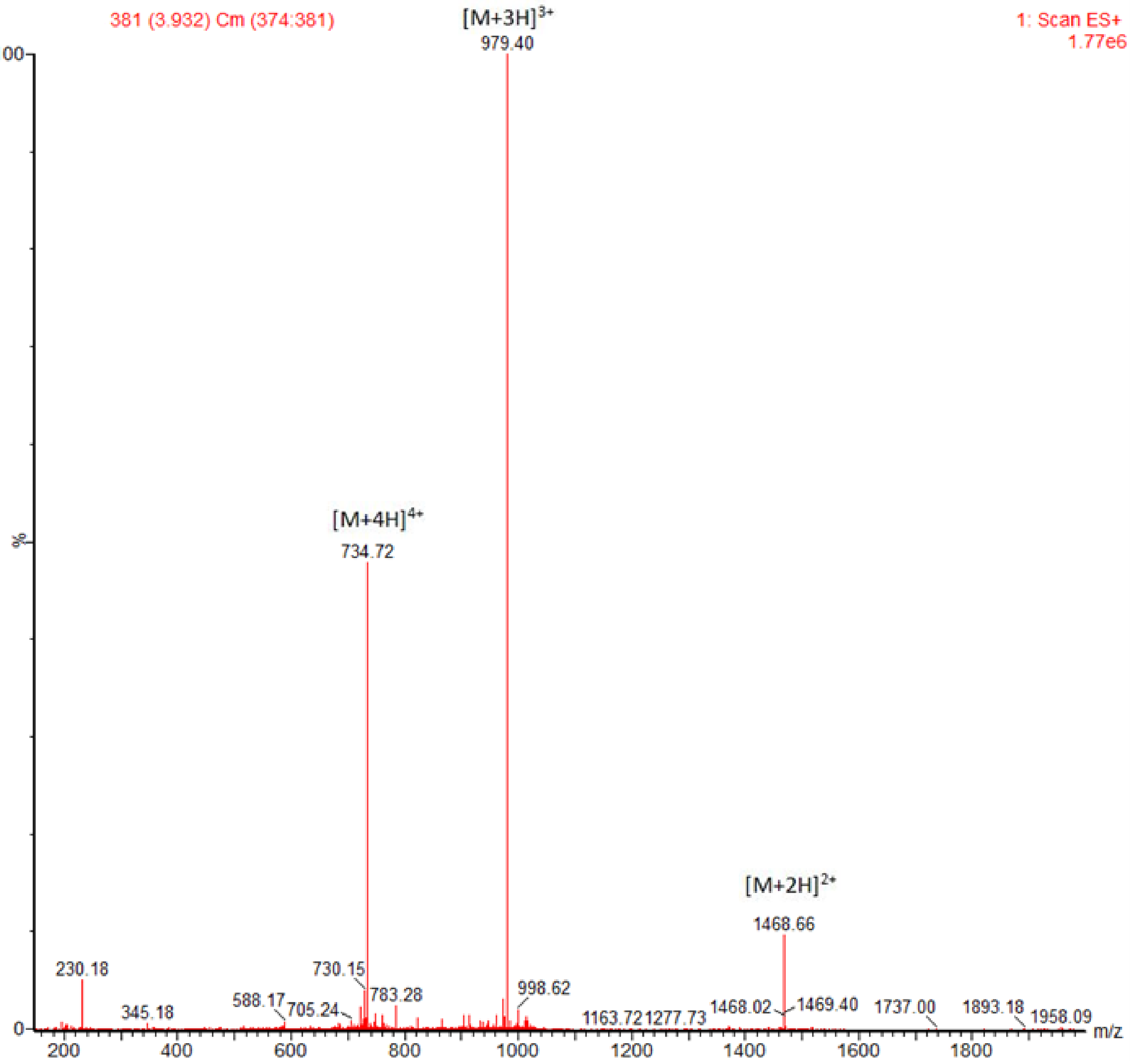

Figure S1. Chromatogram (up) and MS spectrum (down) of MBP (81-106) (**1**).

MBP (76-116) (2)

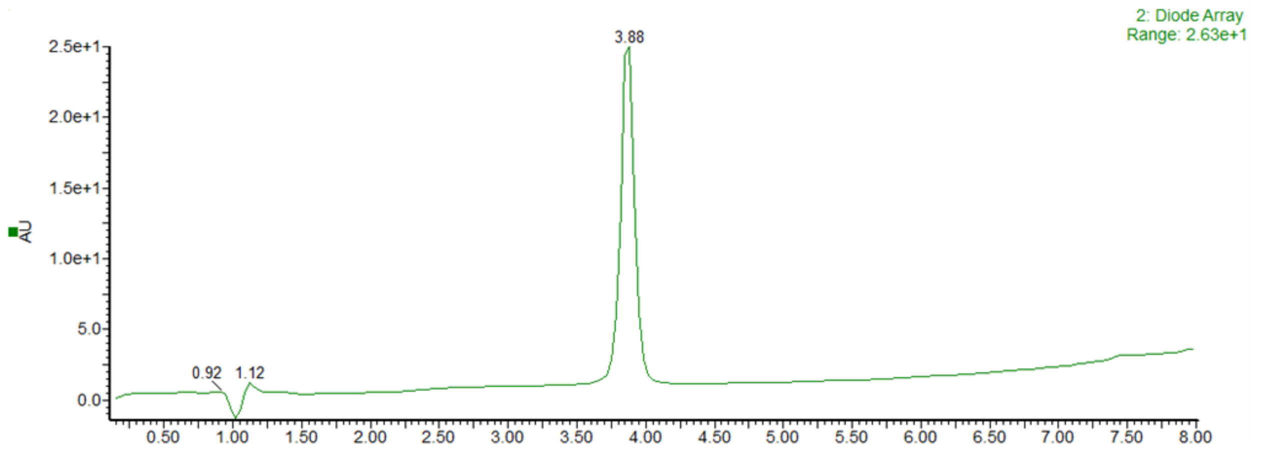

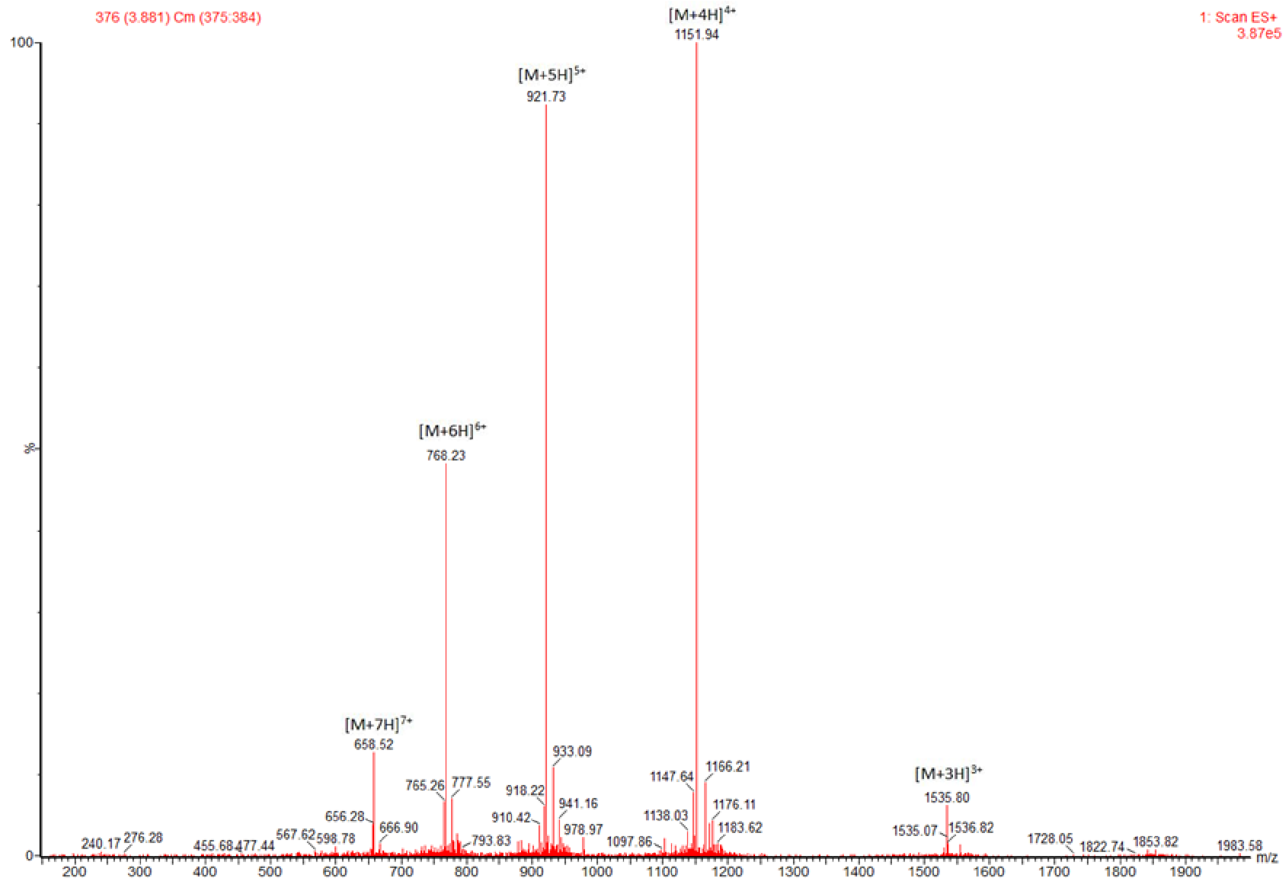

Figure S2. Chromatogram (up) and MS spectrum (down) of MBP (76-116) (**2**).

MBP (76-96) (3)

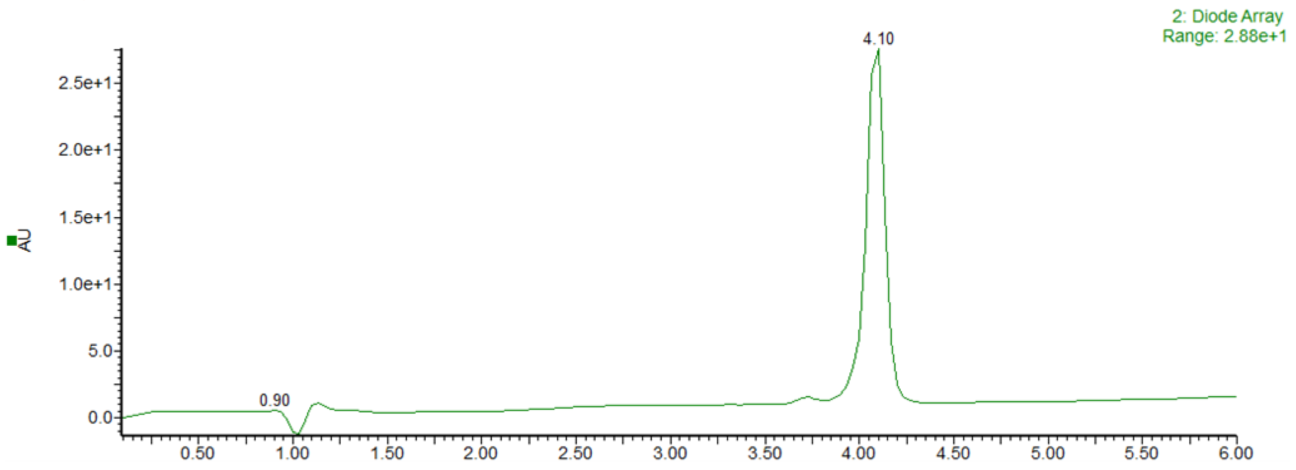

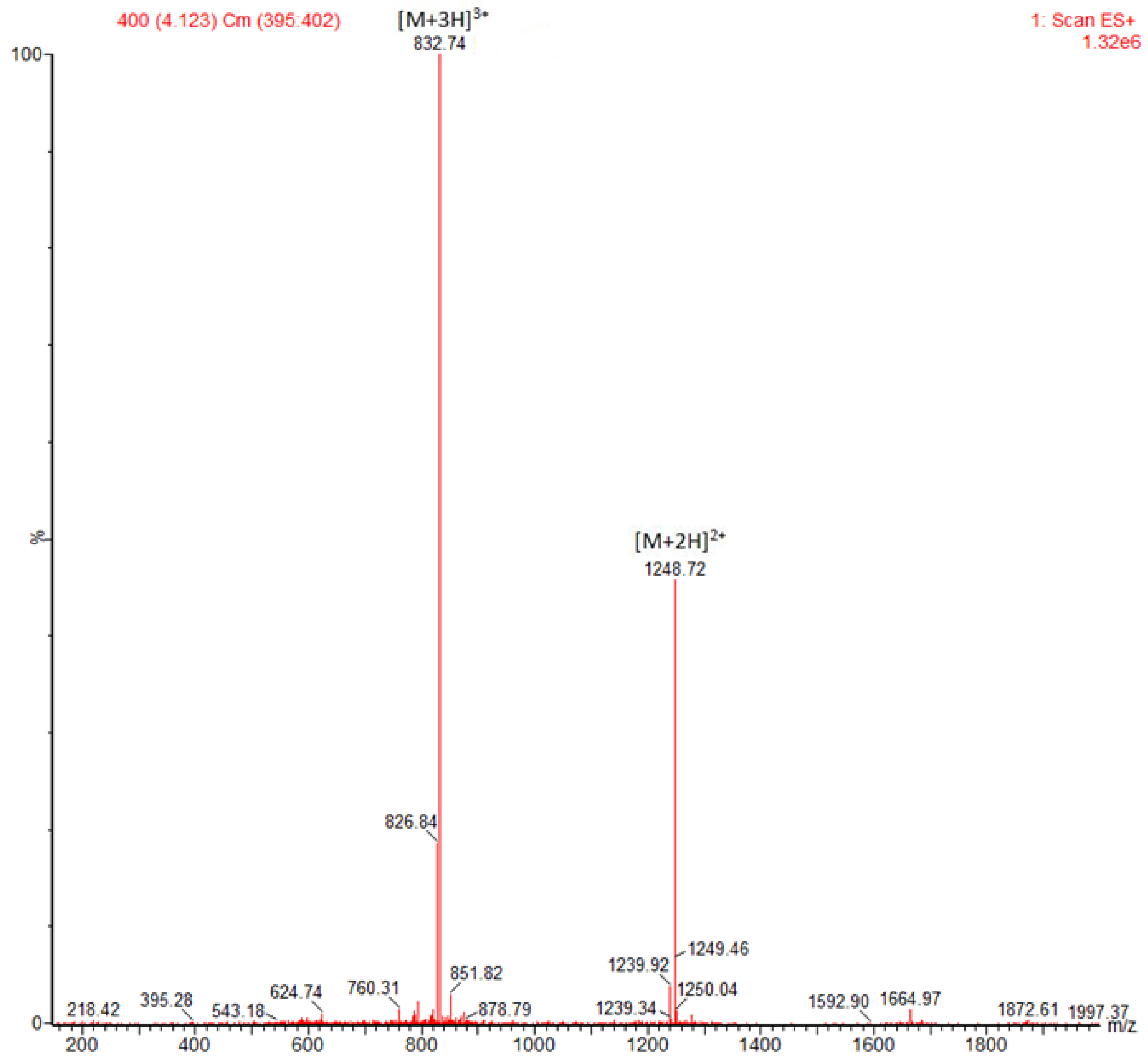

Figure S3. Chromatogram (up) and MS spectrum (down) of MBP (76-96) (**3**).

MBP (97-116) (4)

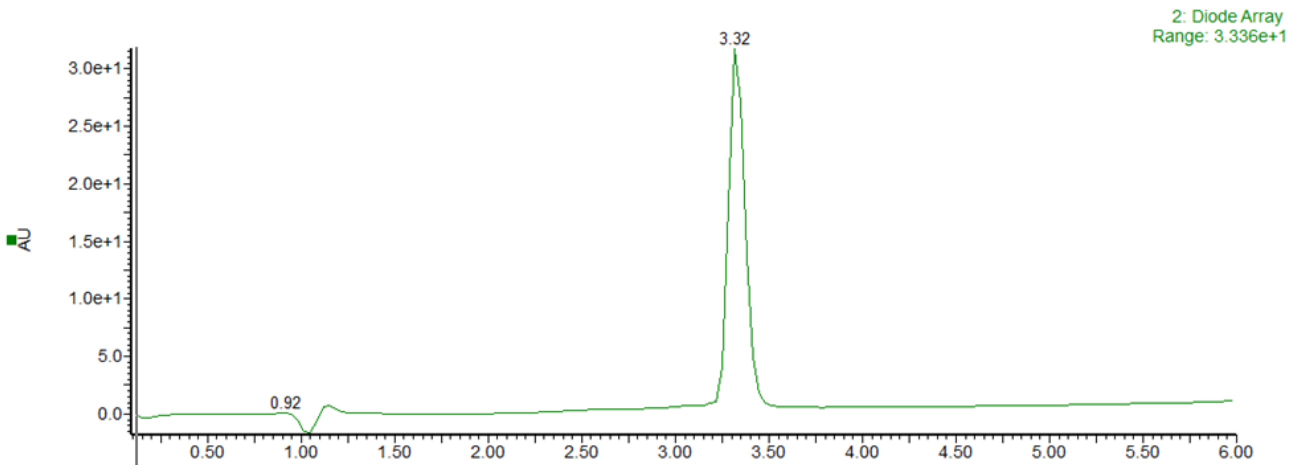

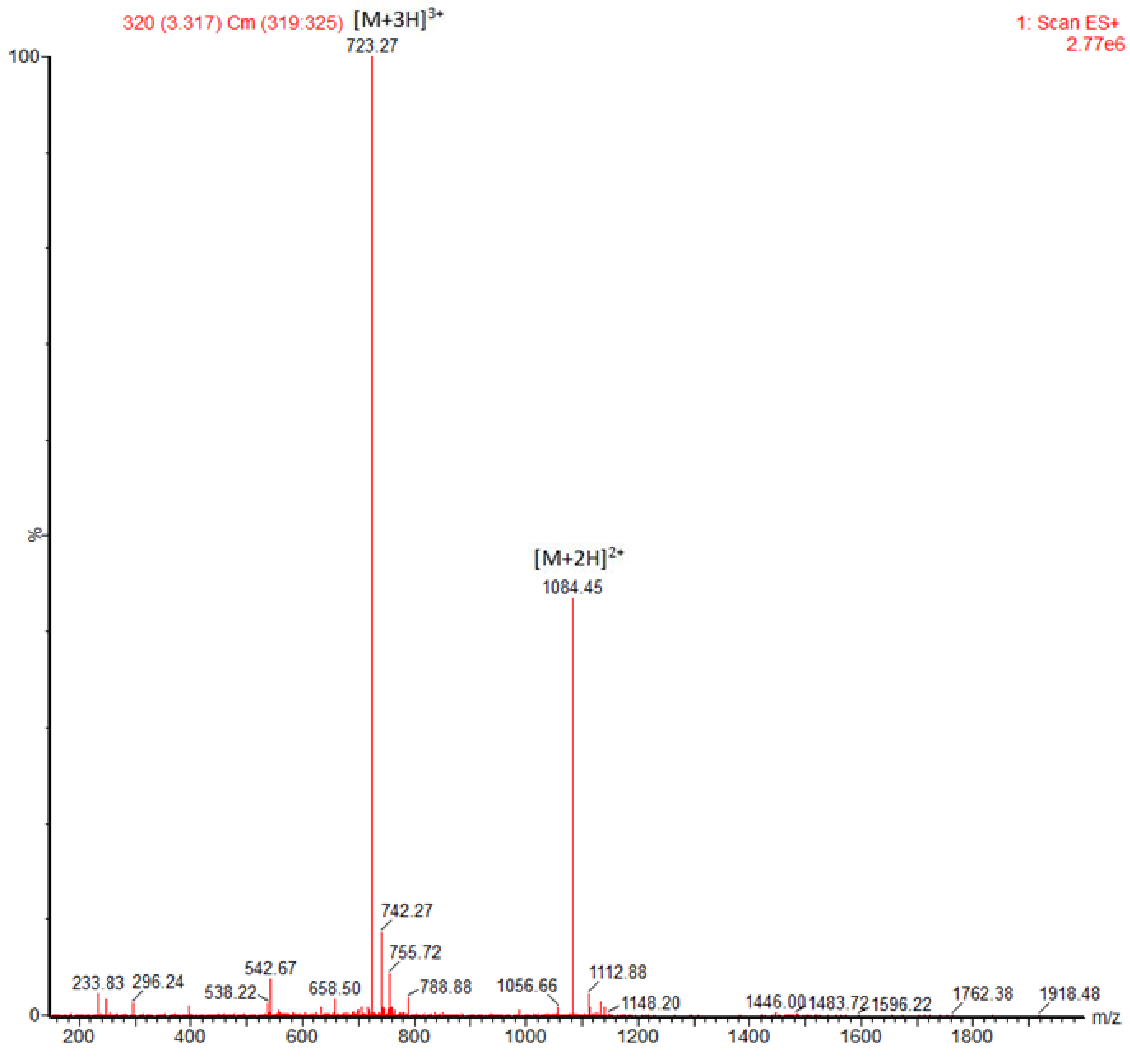

Figure S4. Chromatogram (up) and MS spectrum (down) of MBP (97-116) (**4**)

MBP (81-92) (5)

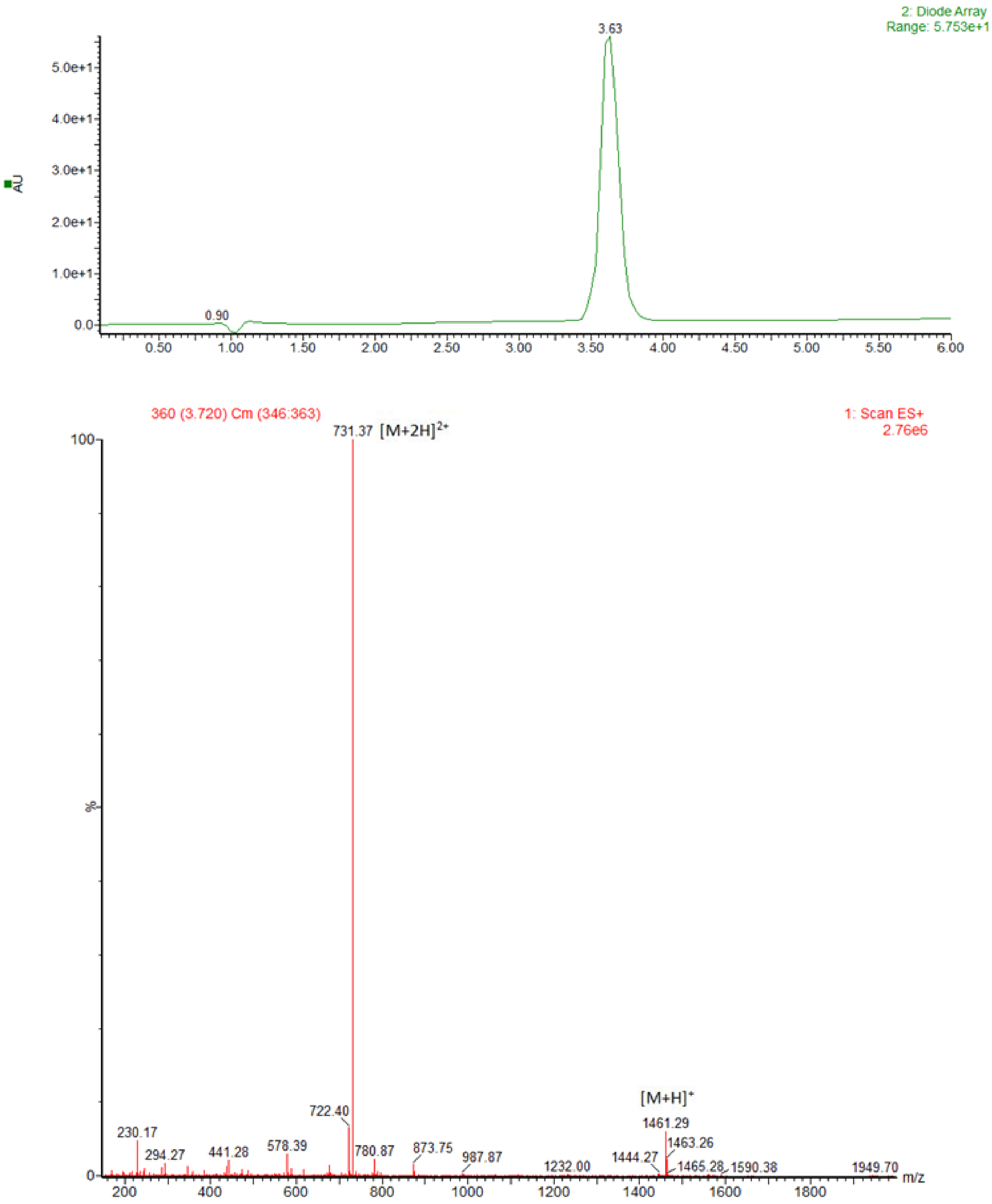

Figure S5. Chromatogram (up) and MS spectrum (down) of MBP (81-92) (**5**).

MBP (99-106) (6)

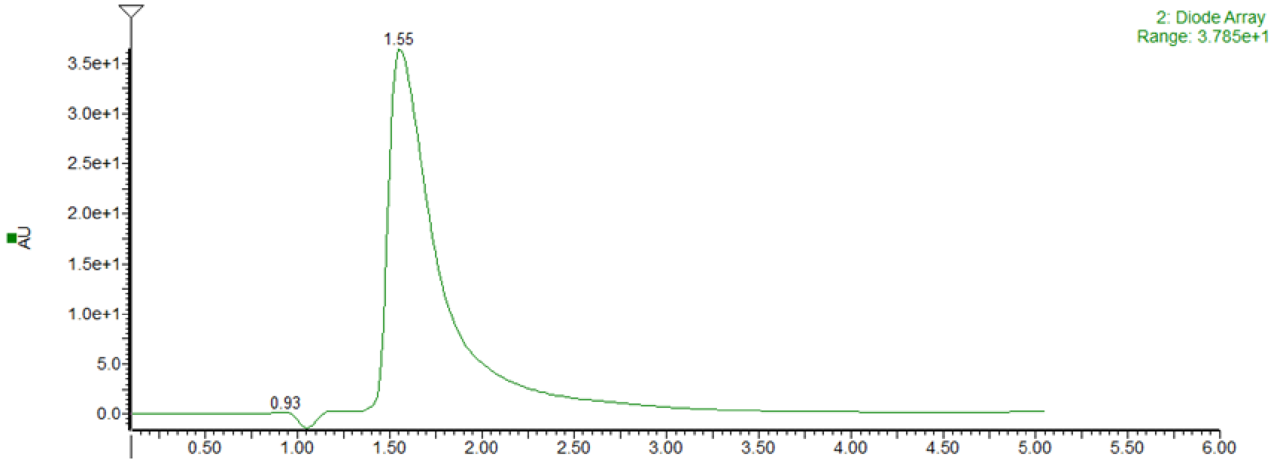

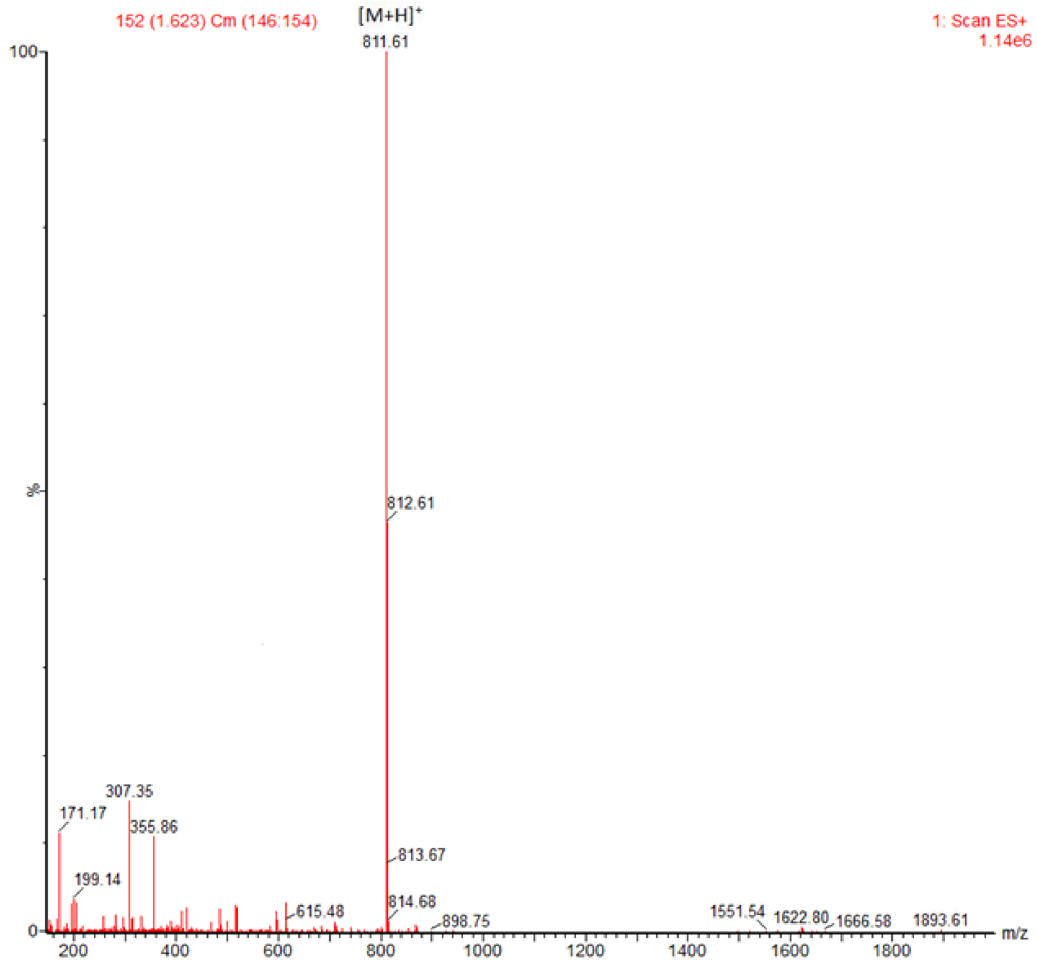

Figure S6. Chromatogram (up) and MS spectrum (down) of MBP (99-106) (**6**).

Set-up of the coating conditions

Peptides were dissolved in buffer carbonate (pH=9.8) or PBS (pH 7.2) independently. Plates were also coated using buffer without peptide to evaluate the influence of peptide in the signals obtained. Polystyrene 96-well ELISA plates were coated with 100 µL/well of a 10 µg/mL solution of synthetic peptide antigens **1-6** diluted in tested buffers. After overnight incubation at 4 °C, plates were washed (3×) using washing buffer. Nonspecific binding sites were blocked with 100 µL/well of fetal bovine serum buffer (10% FBS in washing buffer) or 5% BSA buffer at room temperature for 1 h. Blocking buffer was removed, and plates were incubated overnight at 4 °C with buffer as blank, an expected positive and negative sera (diluted 1:100 in 10% FBS buffer or 2.5% BSA buffer, 100 µL/well). After three washes, plates were treated with 100 µL/well of anti-human IgG or IgM alkaline phosphatase-conjugated specific antibodies diluted in FBS buffer 1:3000 (IgG) and 1:200 (IgM) for all tested antigens. After 3 h of incubation at room temperature and washes (3×), 100 µL of substrate buffer (1 mg/ml *p*NPP, $MgCl_2$ 0.01M in carbonate buffer, pH 9.6) was added to each well. Colorimetric reaction was carried out adding 100 µl of substrate reaction solution (1 mg/ml *p*NPP, $MgCl_2$ 0.01M in carbonate buffer, pH 9.6) to each well and plates were read at 405 nm using a TECAN plate reader. After 30 min, the reaction was stopped with 1 M NaOH solution (50 µL/well) and the absorbance was read in a multichannel ELISA reader (Tecan Sunrise, Männedorf, Switzerland) at 405 nm. Antibody titer values were calculated as (mean Abs of serum duplicate) - (mean Abs of blank duplicate) representing graphically in Figures **S8-S13** the calculated mean values.

		coating buffer 1			coating buffer 2			coating buffer 1			coating buffer 2			
	<>	1	2	3	4	5	6	7	8	9	10	11	12	
peptide coated	A	blank	C+	C-	blank	C+	C-	blank	C+	C-	blank	C+	C-	blocking 1
	B	blank	C+	C-	blank	C+	C-	blank	C+	C-	blank	C+	C-	
	C	blank	C+	C-	blank	C+	C-	blank	C+	C-	blank	C+	C-	blocking 2
	D	blank	C+	C-	blank	C+	C-	blank	C+	C-	blank	C+	C-	
no peptide	E	blank	C+	C-	blank	C+	C-	blank	C+	C-	blank	C+	C-	blocking 1
	F	blank	C+	C-	blank	C+	C-	blank	C+	C-	blank	C+	C-	
	G	blank	C+	C-	blank	C+	C-	blank	C+	C-	blank	C+	C-	blocking 2
	H	blank	C+	C-	blank	C+	C-	blank	C+	C-	blank	C+	C-	
		IgG						IgM						

Figure S7. Samples distribution on the ELISA plate during the set-up of the coating conditions.

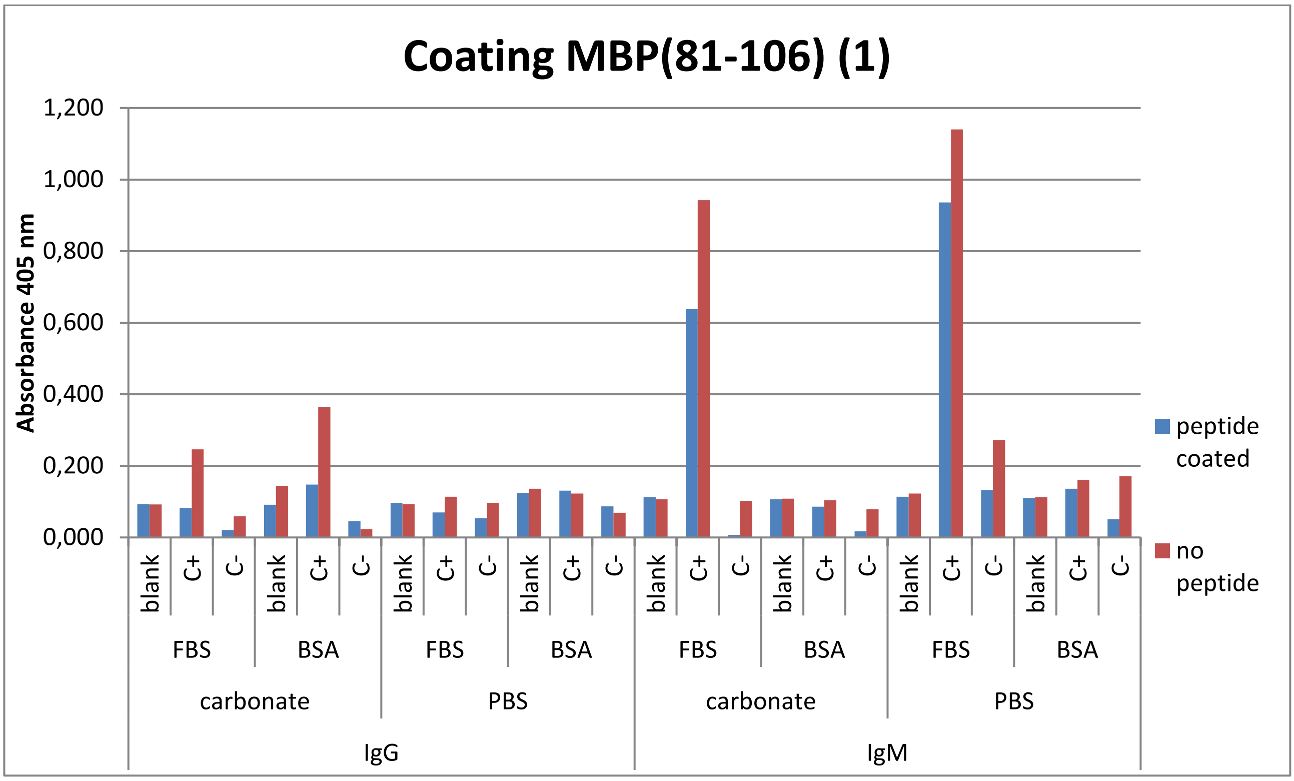

Figure S8. Coating results of Peptide **1**.

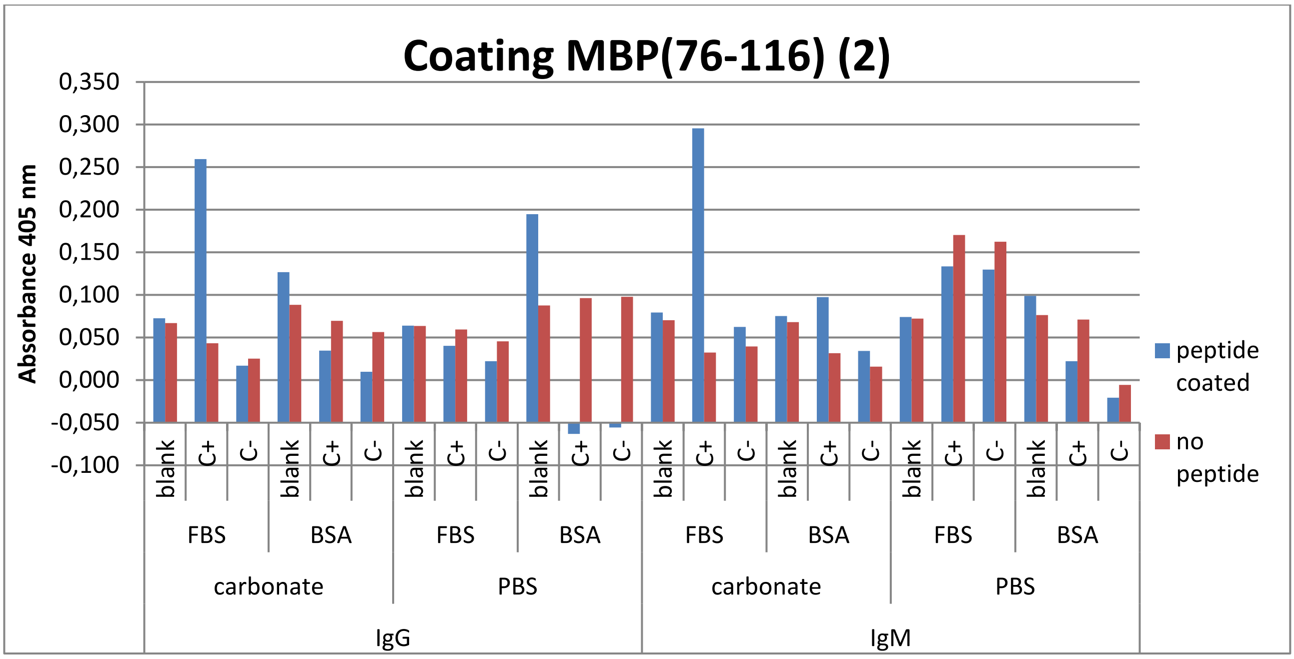

Figure S9. Coating results of Peptide **2**.

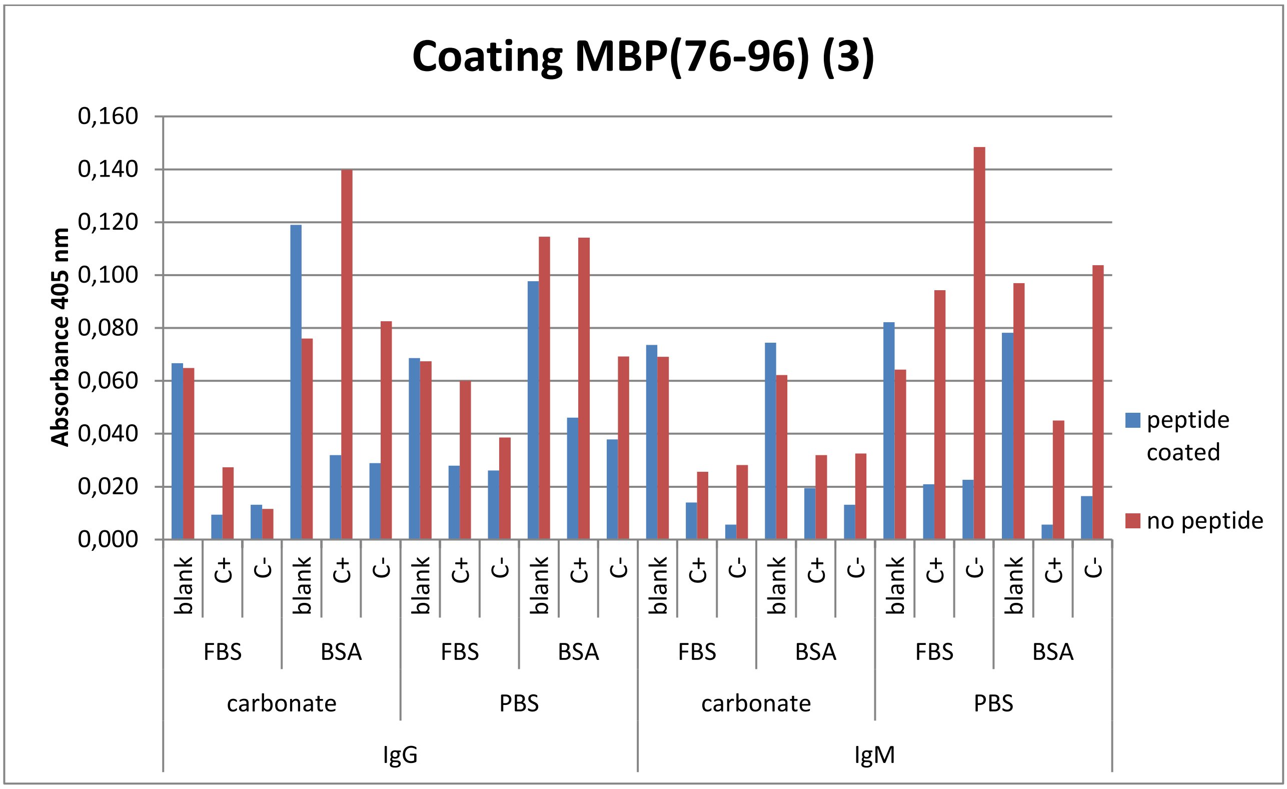

Figure S10. Coating results of Peptide **3**.

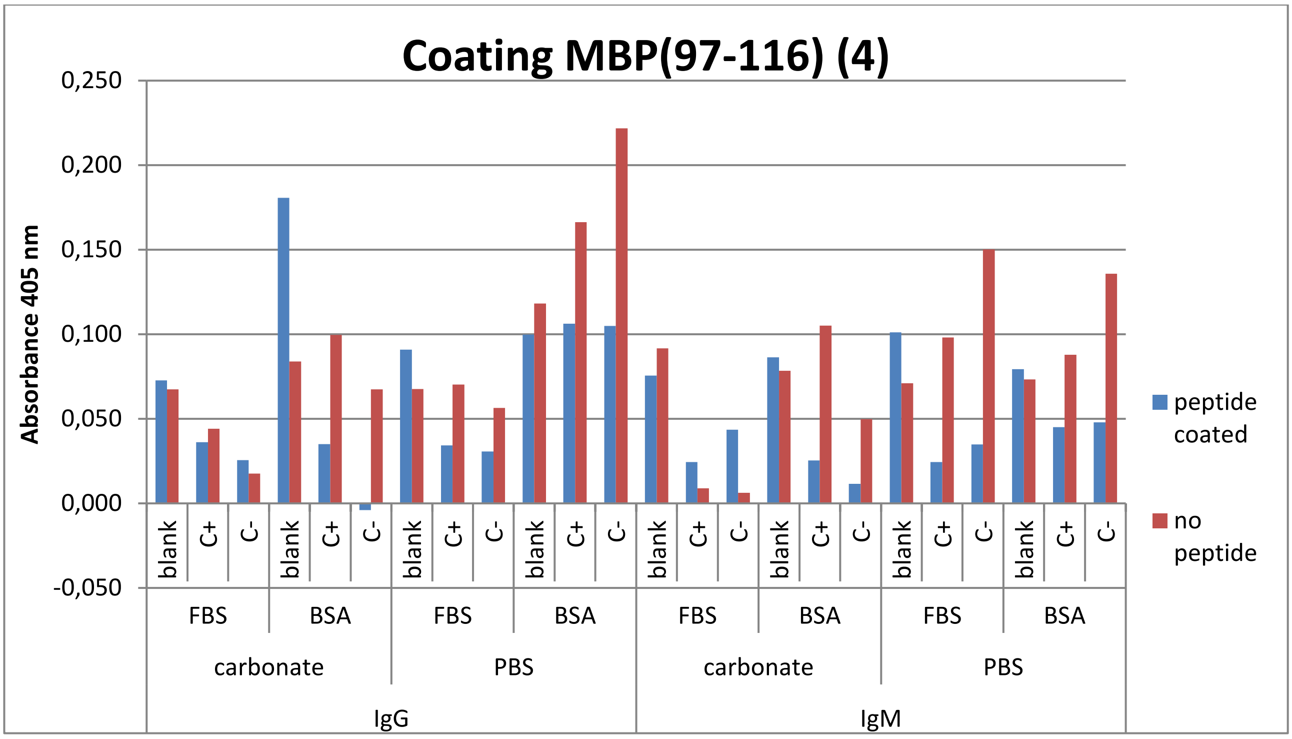

Figure S11. Coating experiments of Peptide **4**.

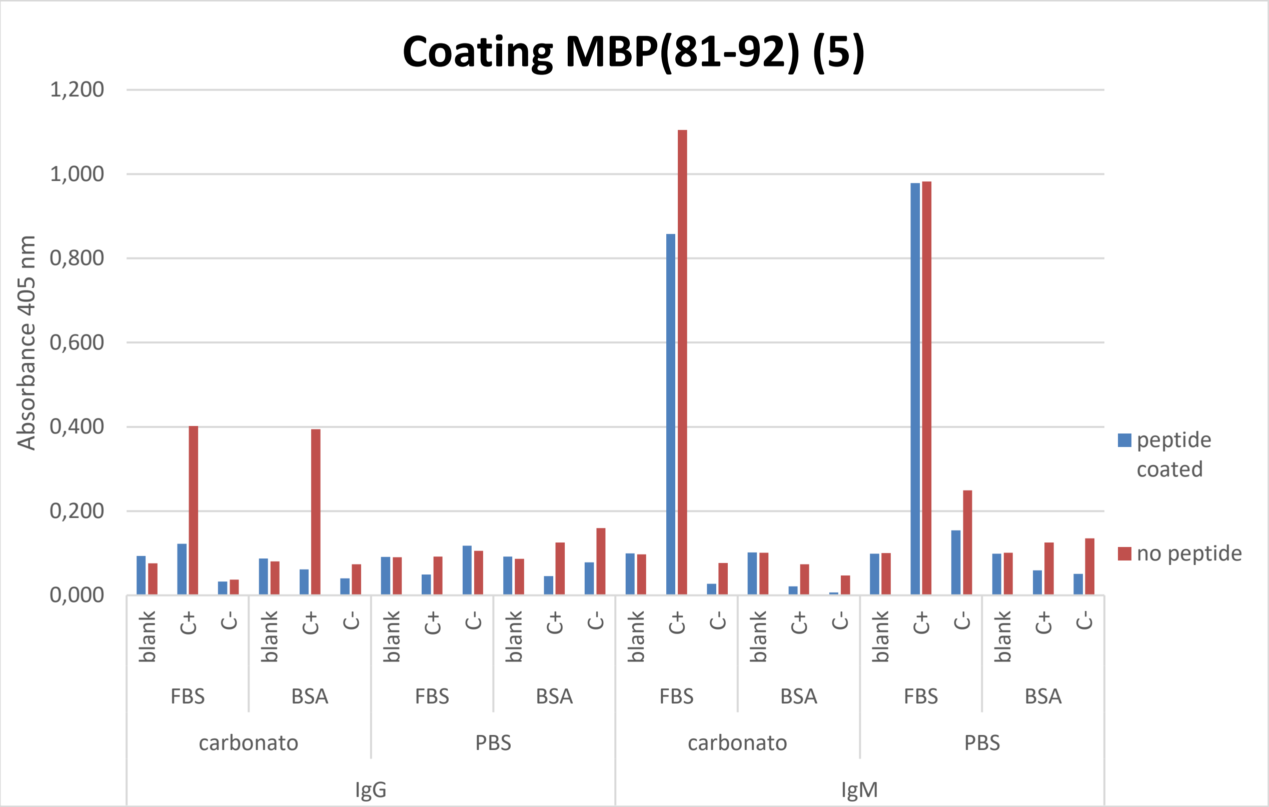

Figure S12. Coating experiments of Peptide **5**.

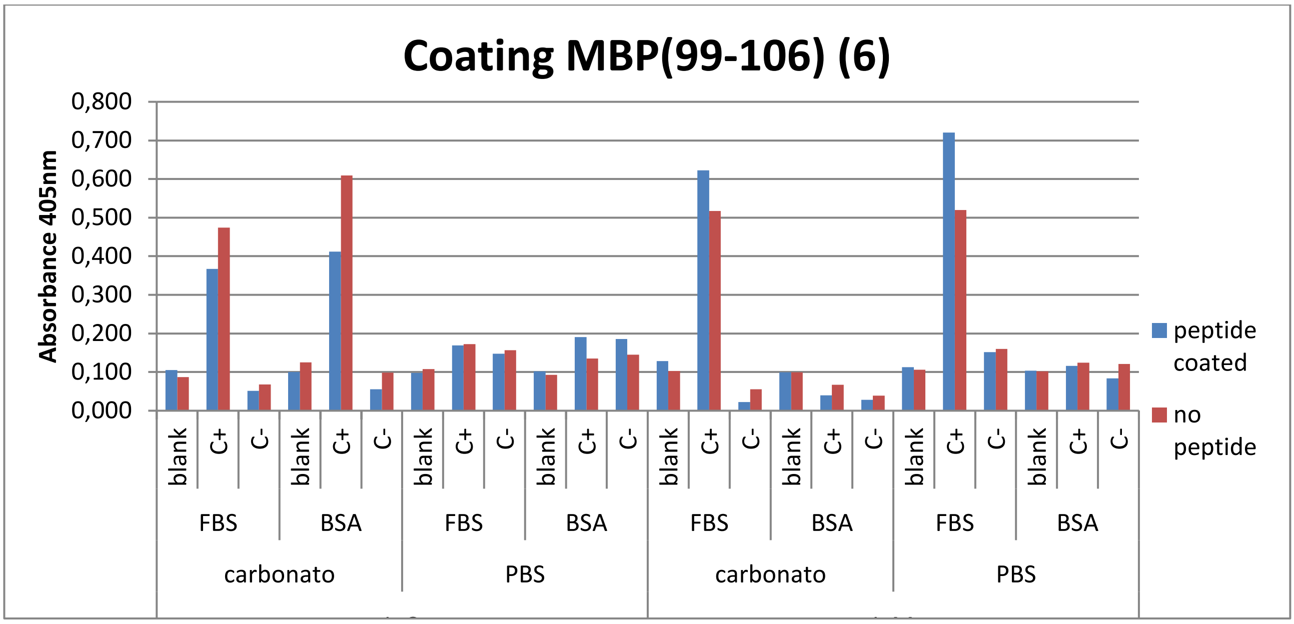

Figura S13. Coating experiments of Peptide **6**.

CD Spectra

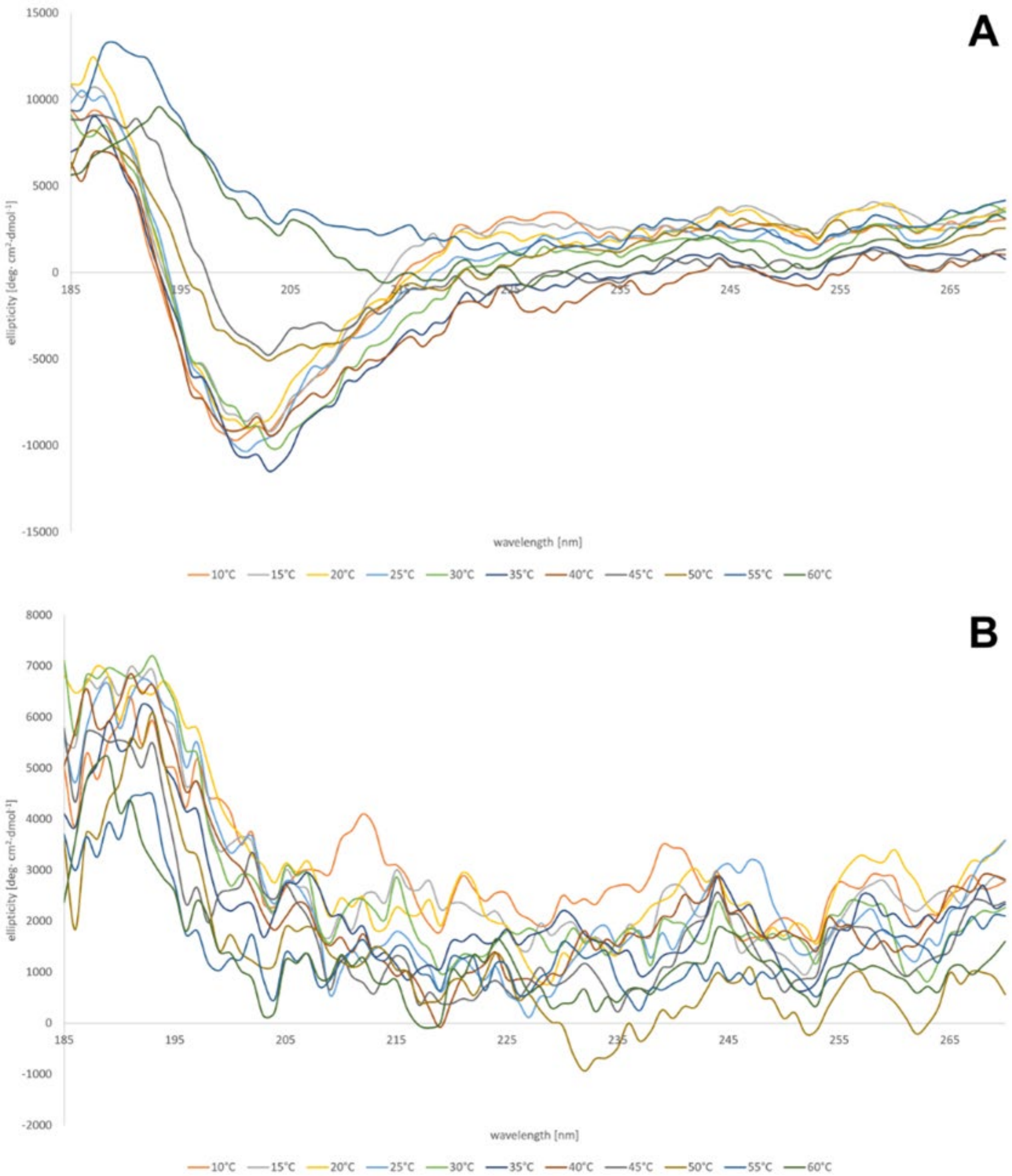

Figure S14. CD spectra registered in water at different temperatures, of the peptides MBP (81-106) (**1**) (**A**) and MBP (76-116) (**2**) (**B**).

CD Spectra

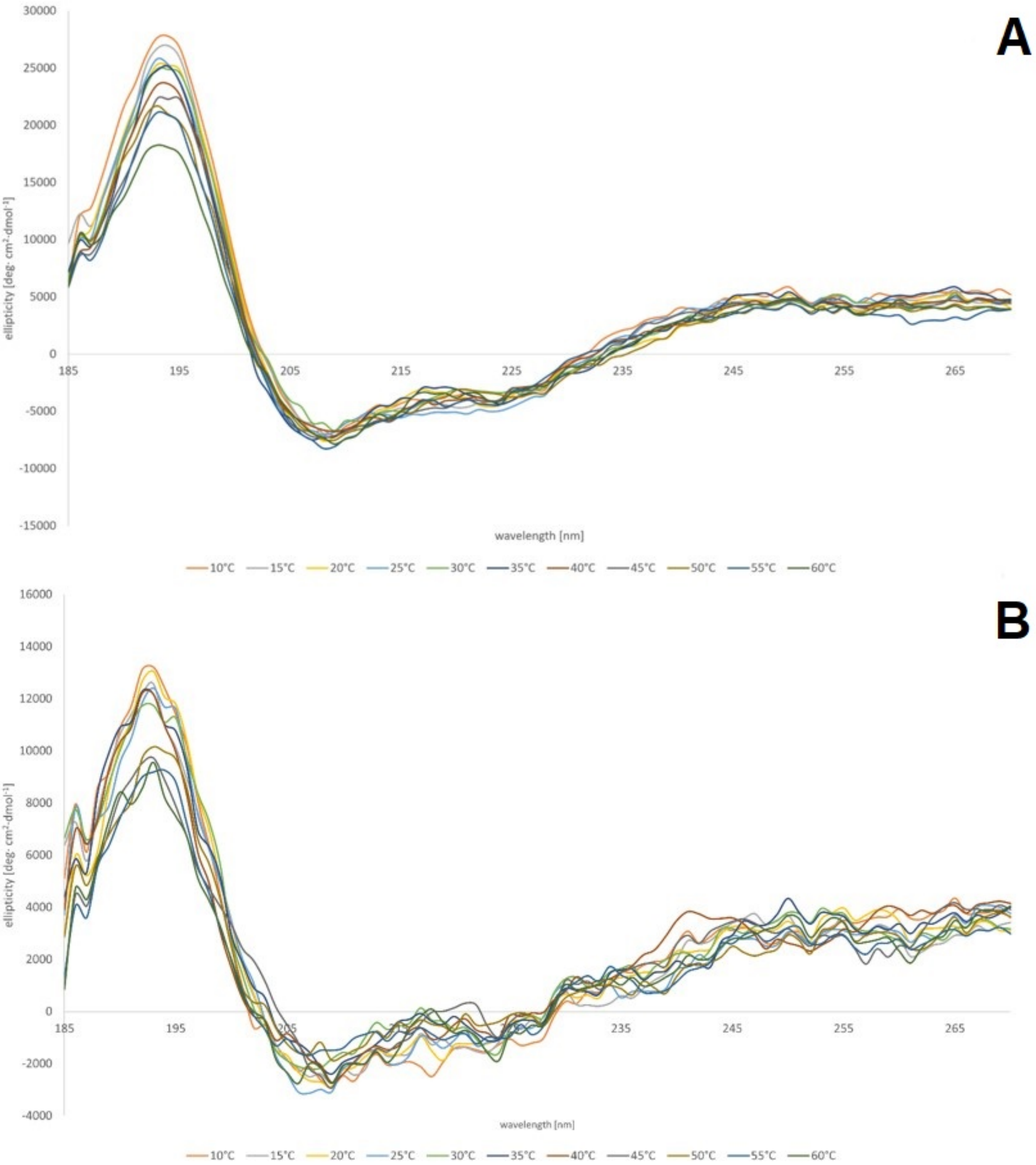

Figure S15. CD spectra registered in H_2O:TFE (50:50, v:v) at different temperatures, of the peptides MBP (81-106) (**1**) (**A**) and MBP (76-116) (**2**) (**B**).

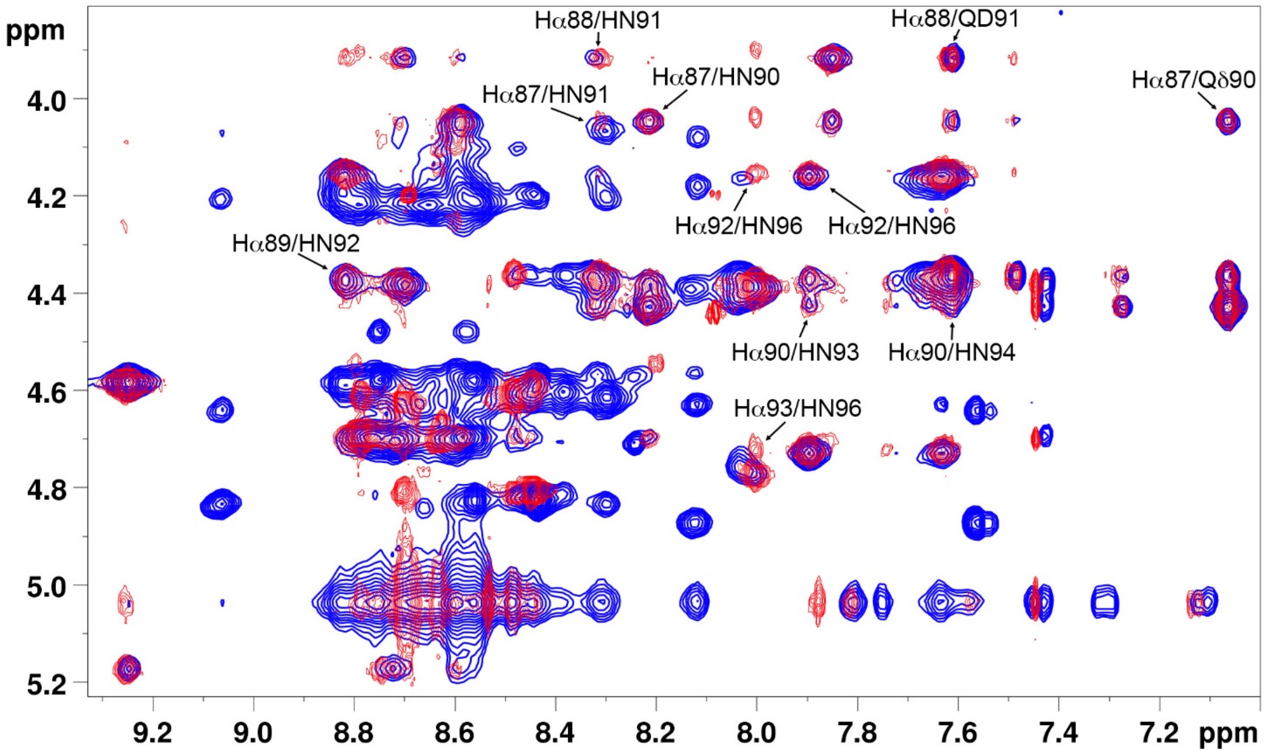

Figure S16. Selected region of the NOESY spectrum of peptides **1** (red) and **2** (blue). Diagnostic signals of the α-helix region are labeled.
